# What Makes Au Nanospheres Superior to Octahedral and Cubic Counterparts for the Deposition of a Pt Monolayer Shell?

**DOI:** 10.1021/jacs.5c03700

**Published:** 2025-07-10

**Authors:** Kei Kwan Li, Lance Kavalsky, Marc Figueras-Valls, Yong Ding, Manos Mavrikakis, Younan Xia

**Affiliations:** † School of Chemistry and Biochemistry, 1372 Georgia Institute of Technology, Atlanta, Georgia, 30332, United States; ‡ Department of Chemical and Biological Engineering, 5228 University of Wisconsin–Madison, Madison, Wisconsin 53706, United States; § School of Materials Science and Engineering, Georgia Institute of Technology, Atlanta, Georgia, 30332, United States; ∥ The Wallace H. Coulter Department of Biomedical Engineering, Georgia Institute of Technology and Emory University, Atlanta, Georgia, 30332, United States

## Abstract

This study demonstrates that Au nanospheres are advantageous over their octahedral and cubic counterparts as seeds in the synthesis of Au@Pt core–shell nanocrystals with a monolayer shell. In combination with experimental characterization, we show through training a machine-learned interatomic potential that the Au nanospheres exhibit a large fraction of low-coordination atoms which are uniformly distributed over the surface. The corresponding high-index facets, including {211}, {311}, {331}, {210}, and {310}, on a spherical seed promote nucleation while greatly shortening the diffusion distance for adatoms. In addition, the high-index facets are instrumental in retaining the deposited Pt atoms on the outermost surface by retarding their inter-diffusional exchange with the underlying Au atoms. By switching from a monolayer made of pure Pt to those made of Pt–Au alloys, we can optimize both the activity and selectivity of the nanocrystals toward the two-electron oxygen reduction reaction for the electrochemical synthesis of H_2_O_2_. This method should be extendible to the fabrication of other core–shell nanocatalysts with desired monolayer shells for various catalytic reactions.

## Graphical abstract

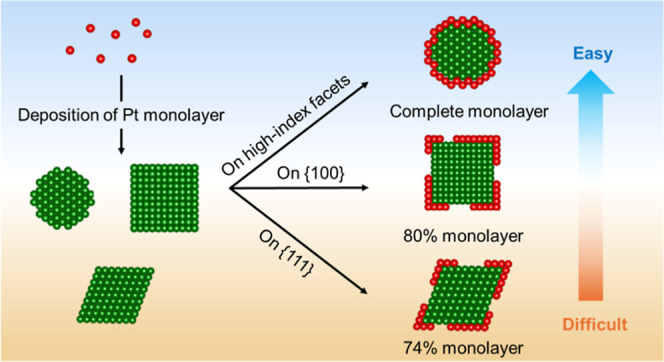

## Introduction

Core–shell metal nanocatalysts (denoted A@B, with the core and shell made of metals A and B, respectively) have garnered significant attention as catalytic materials owing to their enhanced performance toward various reactions, including the oxygen reduction reaction (ORR).^1–6^ Such nanocrystals have been synthesized using a wide variety of approaches, with notable examples involving the co-reduction of two precursors, galvanic replacement, and seed-mediated growth.^7–11^ Seed-mediated growth, in particular, has emerged as the most viable route as it can circumvent detrimental issues such as homogeneous nucleation and destruction of the template, ensuring batch-to-batch reproducibility.^12–16^ In addition, this method also enables the synthesis of core–shell nanocrystals with well-controlled surface structures and compositions, including the formation of bi- and multi-metallic shells, further augmenting their catalytic performance.^17–20^

Significantly, seed-mediated growth has advanced to enable the fabrication of a shell of only one atomic layer in thickness (A@B_1L_).^21,22^ This capability allows one to maximize the atomic utilization efficiency and thus minimize the material cost of a catalyst based on the shell metal. Additionally, the interaction between the core and the shell, through electronic coupling and/or strain effect, can be leveraged to further enhance the catalytic performance. To this end, we have demonstrated that Pd@Pt_*n*L_ nanocubes exhibited the highest mass activity toward ORR at *n* = 1, and the mass activity dropped by threefold when *n* was increased to 6.^21^ In general, the electronic coupling and strain effect would quickly decay as *n* was increased, highlighting the necessity to create a monolayer for the shell in order to realize the maximum potential of the core–shell structure.^23^

Metal nanocrystals with irregular, cubic, octahedral, and icosahedral shapes have all been explored as seeds for the synthesis of A@B_1L_ catalysts in the literature.^21,22,24^ Owing to the presence of a large number of high-index and relatively small facets on the surface, we argue that nanocrystals with a truly spherical shape would be advantageous over other shapes for such a synthesis, but this hypothesis is yet to be validated. First of all, the higher abundance of high-index facets when compared to other shapes makes it possible for the newly formed atoms to nucleate from almost the entire surface of a spherical seed. In contrast, atoms would only nucleate from a limited number of corners (or edges) on the surface of a cubic or octahedral seed.^25–27^ The greater availability of nucleation sites facilitates the formation of a uniform shell on a spherical seed owing to the short distance that the adatoms must diffuse across.^28^ Additionally, as reported in literature,^29^ the high-index facets can mitigate the inter-diffusional exchange between the deposited atoms and those in the core, making it easier to retain the deposited atoms on the outermost surface and thus give a sharper boundary between the core and the shell. The reason why this interesting concept has not been explored in the literature can be attributed to the challenge in synthesizing nanocrystals with a perfectly spherical shape. Most of the so-called “spherical” nanocrystals reported in the literature are either irregular in shape or approximated as truncated icosahedra or cuboctahedra enclosed by {111} and/or {100} facets that are intrinsically more stable than high-index facets.^30–32^ Furthermore, the surface ligands involved in the synthesis tend to favor the formation of low-index facets, making it difficult to access high-index facets.^33,34^

In 2013, a method was reported for the synthesis of single-crystal Au nanocrystals bearing a truly spherical shape (denoted Au nanospheres), together with a uniform and controllable size in the range of 5–150 nm.^35^ Herein, we systematically explore their advantages as seeds for the conformal deposition of a monolayer of Pt by comparing them with Au nanocrystals in cubic and octahedral shapes. Our results from experimental and computational inquiries demonstrate that Au nanospheres enclosed by small high-index facets are superior to their cubic and octahedral counterparts for the creation of a monolayer shell made of Pt. The high-index facets can effectively promote nucleation while mitigating the inter-diffusional exchange between the deposited Pt atoms and the underlying Au atoms. We also extend the synthetic approach to generate monolayer shells made of Pt–Au alloys by co-depositing Pt and Au atoms at different feeding ratios. This capability allows us to optimize the catalytic activity and selectivity of the catalysts toward two-electron ORR for the electrochemical production of H_2_O_2_ by enabling a tight control of the elemental composition on the surface.

## Results and Discussion

### Assignment of the High-Index Facets on Au Nanospheres

We began with the preparation of differently shaped Au nanocrystals (including spherical, octahedral, and cubic) by slightly modifying the protocols reported in the literature.^35–37^ The nanocrystals were then explored as seeds for the conformal deposition of a monolayer of Pt atoms. As shown by the TEM images in Figure S1, the Au spheres were 12.1 ± 0.2 nm and 21.2 ± 0.3 nm in diameter, whereas the octahedra and cubes were 20.1 ± 1.4 and 22.3 ± 0.6 nm, respectively, in edge length. The Au nanocrystals with a size of about 20 nm allowed for a fair comparison to single out the effect of particle shape while the 12 nm Au spheres were interesting for practical catalysis owing to their enlarged specific surface area. We used Fourier-transform infrared (FTIR) spectroscopy to analyze the surface of the nanocrystals. As shown in Figure S1D, all the samples exhibited the doublet peaks at 2900 cm^–1^, corresponding to the stretching modes of CH_2_ group in a long hydrocarbon chain such as cetyltrimethylammonium bromide/chloride (CTAB/C). No additional peaks associated with other species were observed, confirming that all the Au nanocrystals were capped by CTAB/C. As a result, we could rule out any contribution from the difference in surface ligand to the efficacy of Pt deposition. The surface of the octahedral and cubic nanocrystals was supposed to be covered by {111} and {100} facets, respectively, whereas the types of facets on the surface of spherical nanocrystals have not been reported. According to the TEM image, most of the nanospheres had a single-crystal structure without twin defects or stacking faults, ruling out the possibility of having their surface dominated by {111} or {100} facets.

We relied on high-angle annular dark-field scanning transmission electron microscopy (HAADF-STEM) to resolve the types of facets on the surface of the nanospheres. Figure 1A,B, shows the HAADF-STEM images of two 12 nm spheres projected along the [011] and [001] zone axes, respectively, as confirmed by their fast Fourier transform (FFT) patterns (the insets). Apparently, the particles had both {111} and {100} facets on their surface, as marked by the green and red lines, respectively. In addition, the surface contained numerous steps and kinks, as depicted by the purple, yellow, and blue lines. To assign the type of facet to a step or kink, we measured its angle with the (111) or (200) planes, followed by matching the measured angle with the theoretical angle in an ideal FFT pattern corresponding to each zone axis (Figure S2A,B for [011] and [001], respectively). Detailed assignments of the facets are presented in Tables S1 and S2. Specifically, the steps and kinks in Figure 1A could be assigned to {211}, {311}, and {331} facets, while those in Figure 1B corresponded to {210} and {310} facets.

**Figure 1:**
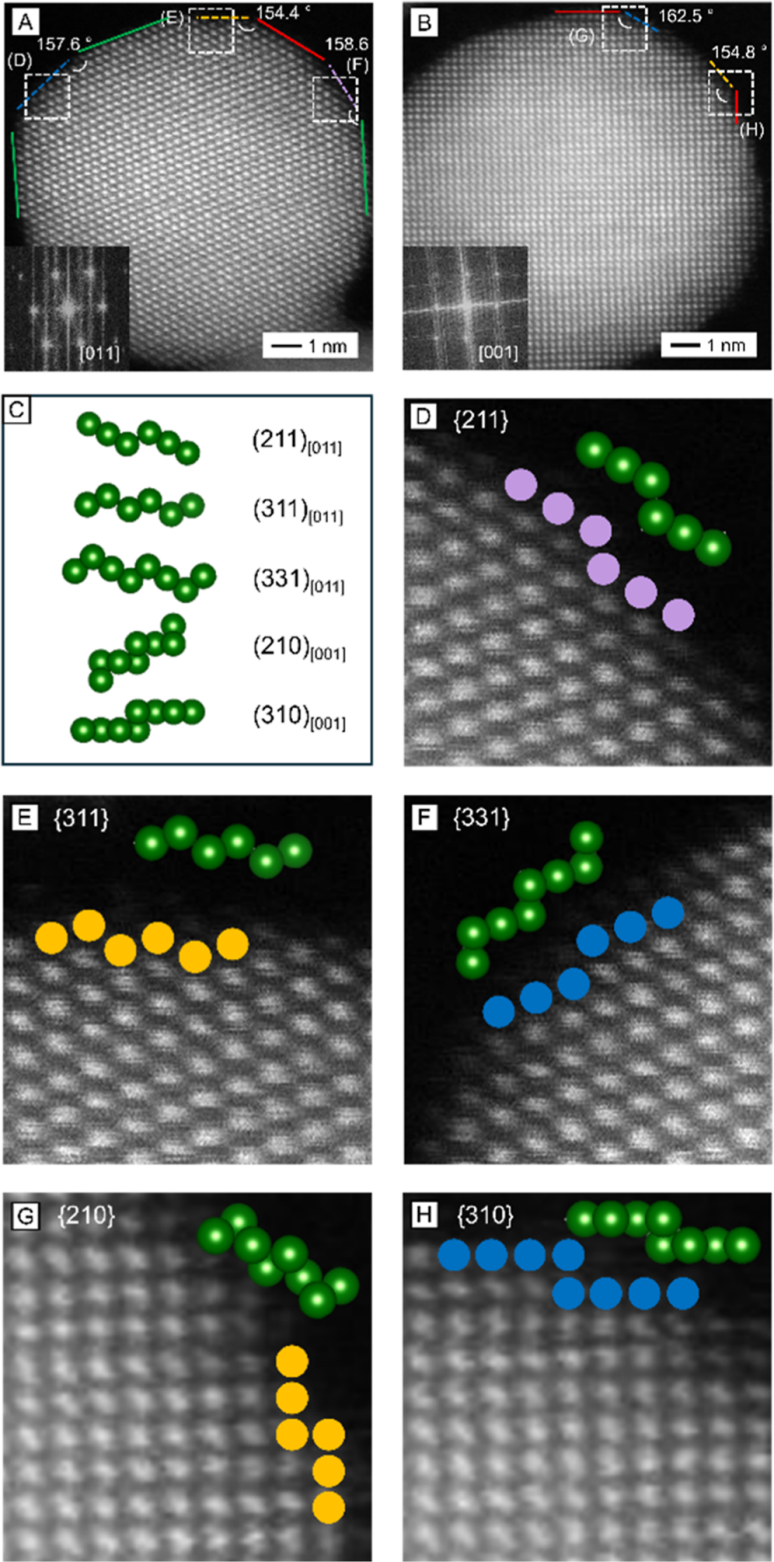
(A,B) HAADF-STEM images of Au nanospheres captured along (A) [011] and (B) [001] zone axes, respectively, together with the assignments of facets on the surface: green = {111}; red = {100}; purple = {211}; yellow = {311} in (A) and {210} in (B); blue = {331} in (A) and {310} in (B), with the insets showing the FFT patterns of the particles. (C) Atomic models of high-index planes identified from the STEM images, viewed along the corresponding zone axes indicated in the subscript. (D–H) Magnified HAADF-STEM images, where steps and kinks were matched to the atomic models in (C) for facet assignments.

To support the facet assignments, we constructed atomic models for each high-index plane viewed along the corresponding zone axes (Figure 1C). These models were then compared with the atomic patterns on the steps and kinks resolved in the magnified STEM images (Figure 1D–H). Overall, the models and the STEM images matched well for the surface atoms, indicating the presence of {211}, {311}, {331}, {210}, and {310} facets on the nanospheres. These five facets are collectively termed high-index facets hereafter. We also constructed an atomic model for the Au nanosphere with the facets identified in the STEM analysis using the VESTA program. When visualized along the [011] and [001] directions (Figure S3A,B, respectively), the model exhibited a nearly perfect spherical shape, which was consistent with the STEM images. To better illustrate the spherical shape, we aligned the model along an arbitrary axis (Figure S3C), allowing all five high-index facets to be observed (marked by red boxes). This model further confirmed that high-index facets were essential for the formation of a truly spherical shape.

In general, STEM images could only be used to provide a conservative estimate of the exposed facets due to two limitations. Firstly, the recorded STEM image was a 2D projection along a specific zone axis, whereas the actual nanosphere should be rounded toward the projection axis in 3D space. As such, the actual indices of the exposed facets were expected to be higher than those assigned above. Secondly, the use of a high-energy electron beam during the characterization might melt the Au nanosphere. The melting would trigger lateral diffusion of surface atoms from high- to low-index facets because of the higher energy associated with the more open structure of a high-index facet.^38^ Although HAADF-STEM analysis is expected to give fewer high-index facets than the real situation, it can be concluded that the surface of a Au nanosphere is covered by a mix of {111}, {100}, and high-index facets, with each one of them bearing a small area.

To address these limitations, computational techniques provide an opportunity for modeling the surface and predicting the presence of high-index facets on the surface at the atomic scale. However, while density functional theory (DFT) calculations can approach chemical accuracy, its computational cost scales cubically with the number of valence electrons in the system, making explicitly modeling Au nanospheres of the size probed in our experiments infeasible. Circumventing this challenge, machine-learned interatomic potentials (MLIPs) are trained on DFT data to predict energies and forces at a fraction of the computational cost.^39,40^ This approach has demonstrated success in modeling systems at length scales previously inaccessible in domains such as heterogeneous catalysis,^41–43^ surface science,^44,45^ and energy storage.^46,47^ For example, MLIPs have been used to model reconstructions of Au surfaces,^48^ as well as the melting of Au nanoparticles,^49^ demonstrating the applicability of this method to Au nanospheres.

Further pushing the frontier of this methodology, recent model architectures incorporating equivariant operations have demonstrated a balance of data efficiency while maintaining state-of-the-art accuracy.^50–52^ These advances have placed emphasis on training set curation that identify information-rich configurations through active learning loops given an initial set of input structures.^53–55^ Thus, in the present work to model these Au nanospheres, we trained an equivariant Allegro potential^52^ on a diverse training set fleshed out via FLARE active learning loops,^54,55^ analogous to recent works.^56,57^ We refer the reader to the Supporting Information for details on training set curation and model training.

Key to the successful application of an MLIP, validation on numerical and physics-guided tests is essential to ensure model accuracy prior to use.^58^ First, we validated the numerical performance of our model on several extended surface facets and nanoparticles (Figure 2A). All numerical validation tests were generated by conducting separate FLARE runs to independently collect information-rich systems for testing (see Table S4 for a complete summary). Test sets with the “internal” label consisted of structures generated by FLARE on input structures that were also used in constructing the training set. We emphasize that these exact test geometries were not explicitly in the training set, but since their corresponding input structures were present, these represent interpolative validation tests. Importantly, these facets in the internal validation set had all been suggested as being present on the Au nanospheres using HAADF-STEM, as discussed above. Across all internal validation tests, root mean squared error (RMSE) values of <1.0 meV/atom were achieved, with the lowest being 0.1 meV/atom for bulk Au and the highest being 0.5 meV/atom for Au(310). Additionally, we observed low RMSE values for both the 147-atom icosahedral Au_147_^ico^ (∼1.6 nm) and 147-atom cuboctahedral Au_147_^cubo^ (∼1.7 nm) nanoparticles. Pushing our MLIP further, tests labeled “external” referred to FLARE sets generated using input structures not in the training set, probing the extrapolative capabilities of the model. Here we considered icosahedral and cuboctahedral systems containing 55 atoms (∼1.1 nm) and 309 atoms (∼2.2 nm), as well as two additional extended step facets (Au(332) and Au(511)). These systems were not included in the training set and posed a greater challenge for our MLIP, with larger RMSE values observed than for the internal validation tests. Interestingly, while the 309-atom (∼2.2 nm) nanoparticles were <10 meV/atom, the MLIP exhibited larger errors for the 55-atom nanoparticles (∼1.1 nm).

**Figure 2:**
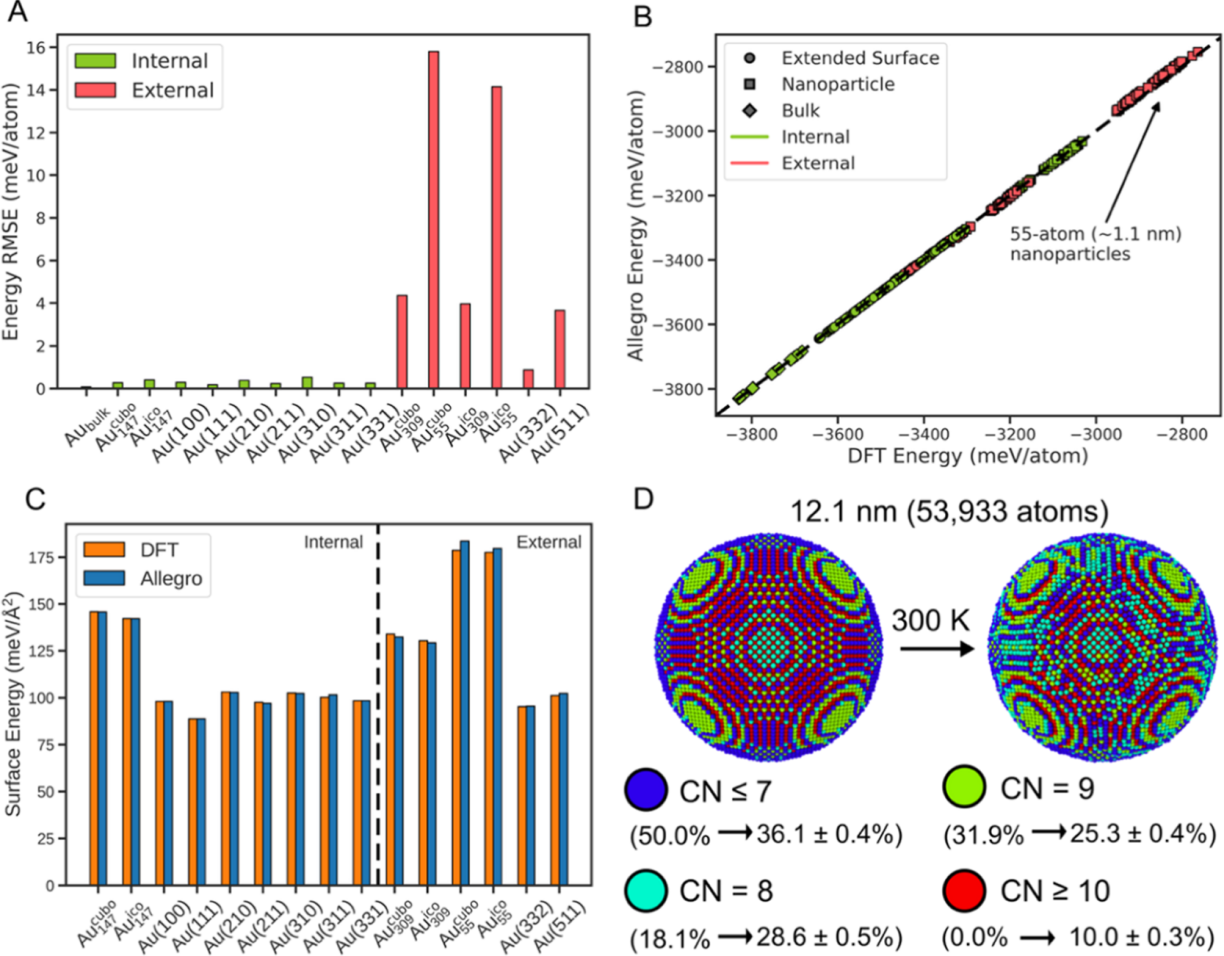
(A) Root mean squared error (RMSE) calculated on internal and external validation tests generated with FLARE. Internal (external) validation tests refer to configurations collected by FLARE where their input structures were (not) used in constructing the training set. Au_x_^cubo/ico^ refers to Au nanoparticles consisting of x atoms in a cuboctahedral/icosahedral geometry. (B) Parity plot of DFT calculated energies and corresponding predictions from our Allegro MLIP. The 55-atom (∼1.1 nm) nanoparticles are highlighted as being out-of-distribution test cases. (C) Comparison of surface energies calculated using DFT and with our Allegro potential. Bars to the left of the dashed line are internal validation tests and bars to the right are external validation tests. Au_x_^cubo/ico^ refers to Au nanoparticles consisting of x atoms in a cuboctahedral/icosahedral geometry. (D) Visualizations of an ideal Au nanosphere (left) and after thermal equilibration +60 ps of MD simulation (right). Atoms are color coded by coordination number (CN). Percentages below the legend indicate fraction present on the surface for the pristine nanosphere structure (left of the arrow) and averaged over the production MD trajectory (right of the arrow).

Rationalizing the relatively weaker accuracy for the smaller nanoparticles, we visualize the parity plot for energy predictions in Figure 2B. On a per-atom basis, the smaller nanoparticles tended to be in a higher energy state than the larger nanoparticles, extended surfaces, and bulk configurations. This finding indicated that these structures may be considered out of distribution in the energy domain, particularly compared to the training set which generally showed Au atoms in lower-energy local environments. However, we anticipated that when scaling the particle size to model the nanospheres, the bulk and extended surfaces would be more representative of the Au local environments, placing more importance on the good performance of our model on those validation tests. Furthermore, with the target of <10 meV/atom, which can be considered a very good fit,^39^ even these weaker predictions did not lie significantly out of this window. Considering the force errors, similar trends to those described for the energy predictions held, and the results are shown in Figure S4.

Besides the numerical performance, we also validated our MLIP on physics-guided validation tests. In this work, capturing the surface energies was core to building confidence in the MLIP’s ability to model the dynamics of the Au nanosphere’s surface. Thus, we calculated the surface energies for all extended surfaces and nanoparticles using both DFT and our trained Allegro MLIP (Figures 2C and S5 plots the errors directly). We refer the reader to the Supporting Information for additional details on the calculation of this quantity. First considering the extended facets, the MLIP could predict the surface energies to within 2.0 meV/Å^2^ with a mean error of 0.4 meV/Å^2^. In terms of capturing the trends in the surface energy, the Allegro MLIP predicted the pairwise differences between surface energies with an average error of 0.4 ± 0.8 meV/Å^2^. Given the average pairwise difference in DFT surface energies across all facets was 4.9 ± 3.8 meV/Å^2^, our MLIP generally captured these thermodynamic trends. In line with the numerical validation, the 55-atom (∼1.1 nm) nanoparticles yielded the largest errors of 4.9 meV/Å^2^ and 2.1 meV/Å^2^ for the cuboctahedral and icosahedral systems, respectively. Furthermore, the 309-atom (∼2.2 nm) nanoparticles exhibited lower errors in predicting surface energies of 1.6 meV/Å^2^ for the cuboctahedron and 1.1 meV/Å^2^ for the icosahedron. Additionally, we calculated energy versus volume for bulk Au and found that our MLIP could predict the DFT equilibrium lattice constant to within 0.07% (Figure S6). As noted earlier, when we scaled up the particle size to 12.1 nm, many of the local environments would approach the limit of Au bulk and surface geometries. Thus, the exceptional performance on predicting bulk properties as well as surface energies, and their trends, for extended surfaces provided confidence in the ability of our Allegro MLIP to accurately model the Au nanospheres. Overall, between the numerical and physics-guided validation tests, our MLIP demonstrated sufficient accuracy for scaling up the system to investigate the Au nanosphere’s surface.

Having established the accuracy of the Allegro MLIP, we simulated a 12.1 nm Au nanosphere consisting of 53,933 atoms, a model at the same scale as our synthesized nanospheres, via molecular dynamics (MD). The calculations enabled simulating the dynamics of the Au nanospheres and estimating the fraction and distribution of surface atoms that could be attributed to the high-index facets. Taking a systematic approach to quantifying the presence of high-index facets, we monitored the CN of the surface atoms. We noted that the surface atoms of crystalline Au­(111) possess a CN of 9 and the surface atoms of Au(100) have a CN of 8. Similarly, we attributed CN ≤ 7 to the steps and kinks present in higher index facets. In addition, Au atoms in the bulk have a CN of 12, and thus we attributed surface atoms with CN ≥ 10 as either being bulk or bulk-like (located just below a high-index facet). From these mappings, coordination analysis allowed us to systematically quantify the percentages of surface atoms associated with terraces and steps. We refer the reader to the Supporting Information for additional methodological details on coordination analysis and identification of the surface atoms.

In Figure 2D on the left, we visualize the pristine nanosphere structure at the start of the MD simulation with surface atoms color-coded by CN. Additional views are shown in Figure S7. On the pristine nanosphere, we observed that there were flat terrace patches (green and blue) with radial rings of small terraces separated by low-CN steps (dark blue) to produce the overall spherical shape. Directly below the surface of these steps, we see higher-coordinated atoms that were bulk-like (red). For this geometry, exactly half of the surface was CN ≤ 7 Au atoms from the concentric steps surrounding small patches of terrace regions.

Applying our trained Allegro MLIP, we simulated the Au nanosphere at 300 K, which was the synthesis temperature. In Figure 2D on the right we visualized the nanosphere at 60 ps after thermal equilibration (120 ps). Over the course of the simulation, the nanosphere maintained its broad spherical shape, however with more Au atoms brought to the surface. This was signified by an increase in the number of atoms identified by the alpha shape method as belonging to the surface,^59^ from 4512 atoms to 5874 ± 16 on average. By bringing these atoms to the surface, the nanosphere was slightly smoothened. Furthermore, we observed a decrease from 50.0% (2256 atoms) to 36.1 ± 0.4% (2120 ± 19 atoms) of the surface having atoms with CN ≤ 7. In tandem, this slightly flattened the steps such that the surface identification algorithm recognizes on average 10.0 ± 0.3% (585 ± 19 atoms) of the surface having a CN ≥ 10 (i.e., exposing the atoms just below the step). Furthermore, highlighting the rounding of the surface, we observed that an overall increase in the total percentage of the surface layer with CN = 8 or 9, which we attributed to relatively flatter regions. However, even with this smoothening of the Au nanosphere’s surface, we note the high percentages of low-CN surface atoms that had a largely uniform distribution over the surface, a property unique to the nanosphere geometry.

### Deposition of a Pt Monolayer on Au Nanospheres

These small areas intrinsic to the facets on Au nanospheres made them ideal substrates for the conformal deposition of a monolayer made of a different metal due to the short distance needed for the adatoms to diffuse across. The higher abundance of high-index facets also made them advantageous over octahedral and cubic counterparts in the fabrication of a monolayer comprised of another precious metal such as Pt for two main reasons. Firstly, the high surface energies inherent to high-index facets made them favorable for the nucleation and deposition of newly-formed Pt atoms. Secondly, the high-index facets could help retain the deposited Pt atoms on the outermost layer of a nanosphere, as confirmed by a computational study (see below).

We coated the surface of the Au nanocrystals of different shapes with Pt atoms by reducing PtCl_4_^2–^ with an excess amount of ascorbic acid (AA) at 27 °C in an aqueous system. We chose Pt for investigating the deposition process owing to the relatively high reduction potential of its precursor (e.g., PtCl_4_^2–^ at 0.75 V), making it reducible by AA at 27 °C. At this relatively low temperature, the undesired inter-diffusional exchange between the Pt atoms in the shell and the Au atoms in the core was minimized. As observed in our recent study, Au cubic nanocrystals were prone to shape transformation even at room temperature due to the diffusion of Au atoms from the corners to the {100} facets which helped reduce the total surface energy of the particles.^28^ A similar transformation also occurred during the deposition of Pt monolayer in the current work and the final products were found to take a cuboctahedral shape encased by a mix of {100} and {111} facets. Despite this transformation, the sample is still referred to as cubic nanocrystals in our discussion to reflect the original shape of the seeds.

By controlling the amount of PtCl_4_^2–^ added relative to the dimensions and concentration of the seeds, we aimed to deposit a Pt monolayer on the Au nanocrystals of different shapes. For simplicity, the resultant particles are denoted Au@Pt_1L_ hereafter. Figure 3A shows a typical TEM image of the sample involving 12 nm Au spheres, indicating that the spherical shape, as well as size uniformity, was preserved due to the involvement of a uniform and ultrathin Pt shell. For the same reason, the average diameter of the spheres was largely maintained at 12 nm. We also used inductively-coupled plasma mass spectrometry (ICP-MS) analysis to confirm that a Pt monolayer was indeed formed. The atomic ratio of Pt to Au in the Au@Pt_1L_ nanospheres was determined to be 6.2:93.8, corresponding to ca. 1.09 atomic layer of Pt on the surface of each Au nanosphere based on a diameter of 12 nm for the spherical seeds (Table 1). Moreover, the HAADF-STEM image and the corresponding magnified image taken from a Au@Pt_1L_ nanosphere along the [011] zone axis (Figure 3B,C) confirmed the presence of both {111} and {100} facets on the surface, together with steps and kinks. The steps and kinks could be assigned to {211}, {311}, and {331} facets, consistent with the surface structure of the original Au nanosphere. The capability to retain the high-index facets could be attributed to the deposition of a uniform Pt monolayer, which should not cause alteration to the surface structure of the underlying nanosphere.

**Figure 3:**
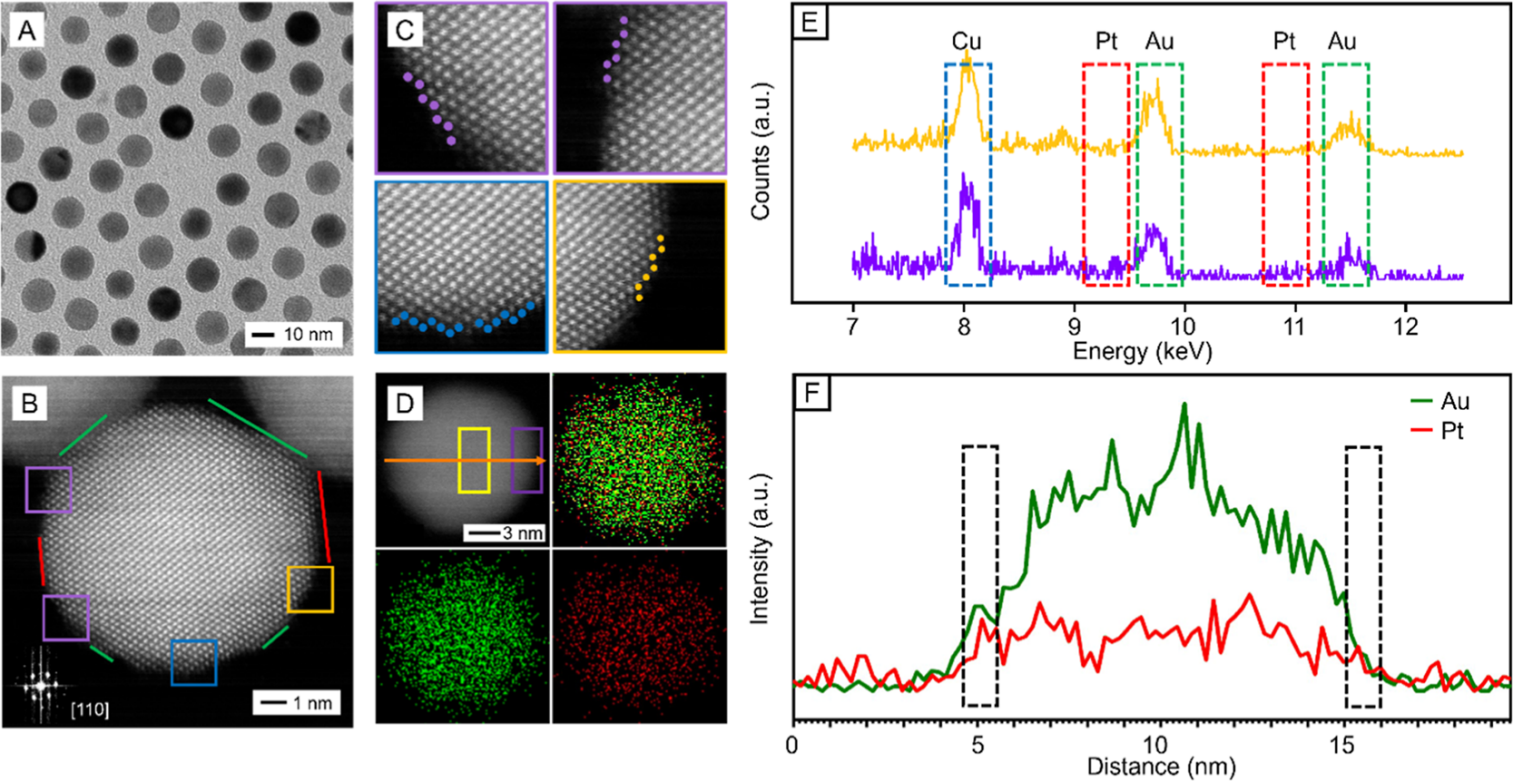
Characterizations of a 12 nm Au@Pt_1L_ nanosphere (A) TEM image; (B) HAADF-STEM image along the [011] zone axis, with the inset showing an FFT pattern of the particle; (C) magnified HAADF-STEM images, together with the assignments of high-index facets on the surface: purple = {211}; yellow = {311}; and blue = {331}; (D) HAADF-STEM image and the corresponding EDX mapping (red: Pt; green: Au); (E) EDX spectra of the two regions boxed in (D); and (F) EDX line-scan of a Au@Pt_1L_ nanosphere along the orange arrow in (D), indicating that the Au and Pt signals shared the same intensity at the edges of the particle (dash boxes).

**Table 1:** Elemental Compositions (Pt to Au Atomic Ratios) When We Attempted to Deposit a Pt Monolayer on the Surface of Au Nanocrystals of Different Shapes and Sizes^a^

sample	Pt to Au ratio from ICP-MS	standard deviation of the Pt content (%)	actual *n* in Au@Pt_*n*L_
12 nm Au@Pt spheres	6.2:93.8	±0.1	1.09
21 nm Au@Pt spheres	3.7:96.3	±0.5	1.06
20 nm Au@Pt octahedra	4.7:95.3	±1.1	0.58
22 nm Au@Pt cubes	4.4:95.6	±1.2	0.77

a The time for reactions were kept the same for fair comparison.

We further employed energy-dispersive X-ray spectroscopy (EDX) mapping and line-scanning to characterize the spatial distribution of Pt and Au atoms in a Au@Pt_1L_ nanosphere. As shown in the EDX mapping (Figure 3D), the Pt signal was distributed uniformly across the particle, while the Au signal was mainly confined to the core. This pattern was consistent with what was observed for Pd@PtIr_1.5L_ icosahedral nanocrystals.^22^ The even distribution of Pt signals suggested that the Pt atoms were predominantly located on the periphery of the particle, with minimized inter-diffusional exchange with the Au atoms underneath. Otherwise, the Pt signal would spread into the core, imitating the Au distribution. Additionally, the absence of clustering for the Pt adatoms eliminated the possibility of uneven Pt deposition. Overall, the EDX mapping confirmed the presence of Pt atoms on the outermost surface of the Au nanosphere, together with negligible inter-diffusional exchange with the core.

We also analyzed the EDX spectra in different regions of the Au@Pt_1L_ nanosphere (Figure 3E), as marked by the purple and yellow boxes in Figure 3D. These two regions correspond to the surface and the core of the particle, respectively. The EDX spectrum recorded from the surface region (purple trace) featured Pt peaks with higher intensity around 9.5 and 11 keV when compared to that from the core (yellow trace). The difference in intensity implied a higher Pt content on the surface than in the core, suggesting that the inter-diffusional exchange between the Pt atoms in the shell and the Au atoms in the core was largely suppressed. It should be noted that we normalized both the EDX spectra with respect to the Cu signal from sample grid for fair comparison. Furthermore, EDX line-scan (Figure 3F) along the orange arrow in Figure 3D revealed that the Pt and Au signals had similar intensities in the outermost region of the Au@Pt_1L_ nanosphere (marked by the dash boxes). The data supported that the Pt atoms remained in the near-surface region because the inter-diffusion between the Pt adatoms and underlying Au atoms was mitigated to a large extent. Otherwise, a Au-rich surface would be formed and a significantly higher Au intensity would be observed when compared to Pt in the dash-boxed region. In addition, the Pt signal showed a steady intensity across the Au@Pt_1L_ nanosphere, further confirming that the Pt atoms remained in the near-surface region.

The results from ICP-MS, HAADF-STEM, and EDX analyses suggested the formation of a Pt monolayer shell on the surface of each Au nanosphere. However, the similar atomic number and *Z* contrast between Au and Pt made it difficult to directly resolve the core–shell structure using STEM. To address this issue, we measured the distances between neighboring Pt atoms in the shell and neighboring Au atoms in the core. Specifically, we plotted the atomic intensity profiles along the red and green arrows in Figure 4A, corresponding to the first and second atomic layers, respectively, of a Au@Pt_1L_ nanosphere along the [111] direction. For comparison, we also acquired similar plots from a Au nanosphere along the blue and pink arrows in Figure 4B, with the results color-coded and summarized in Figure 4C. We then measured the interatomic distance across 5 atoms in each plot. In the case of Au nanosphere, the first and second layers exhibited nearly identical spacings of 1.24 and 1.23 nm, respectively (blue and pink traces). In contrast, the first layer of the Au@Pt_1L_ nanosphere gave a shortened spacing of 1.18 nm (red trace), whereas the second layer remained at 1.24 nm (green trace). Given that Pt has a smaller atomic size than that of Au, the shortening of atomic distance in the outermost layer of Au@Pt_1L_ nanosphere confirmed the formation of a Pt monolayer on Au nanosphere.

**Figure 4:**
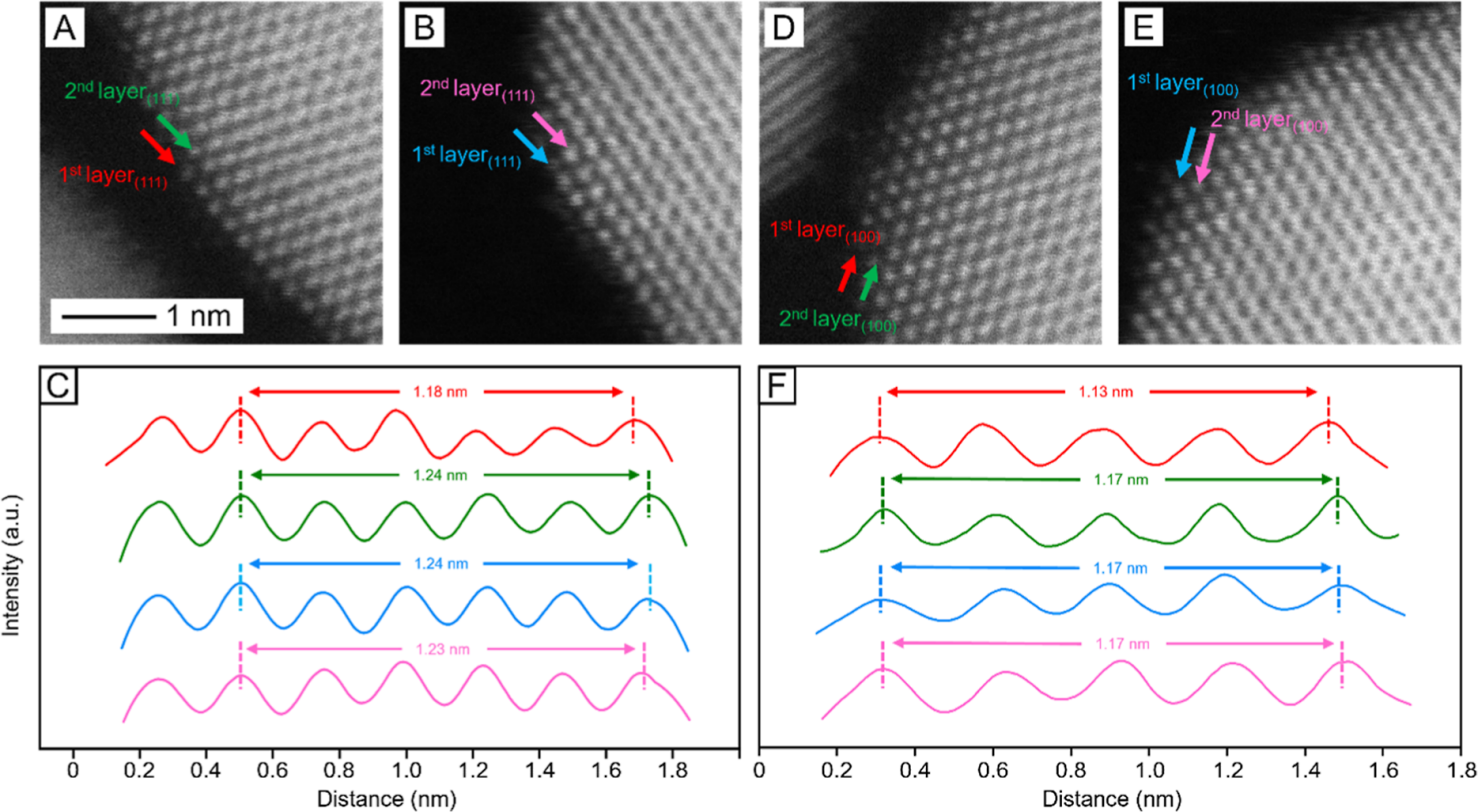
(A,B) HAADF-STEM images of a (A) Au@Pt_1L_ and (B) Au nanosphere. (C) Atomic intensity profiles measured along different arrows in the STEM images (red and green: first and second layer of Au@Pt_1L_ nanosphere along the [111] direction, respectively; blue and pink: first and second layer of Au nanosphere along the [111] direction, respectively). (D,E) HAADF-STEM images from different regions of the same (D) Au@Pt_1L_ in (A,E) Au nanosphere in (B). (F) Atomic intensity profiles measured along different arrows in the STEM images (red and green: first and second layer of Au@Pt_1L_ nanosphere along the [100] direction, respectively; blue and pink: first and second layer of Au nanosphere along the [100] direction, respectively).

We repeated the measurement along the [100] direction on the same Au@Pt_1L_ and Au nanospheres (Figure 4 D–F), but in different regions. For the Au nanosphere, both atomic layers had a spacing of 1.17 nm across 4 atoms (blue and pink traces). Meanwhile, the Au@Pt_1L_ nanosphere showed a shortened spacing of 1.13 nm in the first layer (red trace), consistent with the observation along the [111] direction. Taken together, the interatomic distance measurements along both [111] and [100] directions confirmed the presence of a Pt monolayer on Au nanosphere.

To validate the deposition of a complete Pt monolayer, we measured the interatomic distances at multiple sites on the same Au@Pt_1L_ nanosphere in Figure 4A,D, as circled in Figure S8. The average spacings, together with standard deviations, are summarized in Table S5. These values matched with those obtained from the regions highlighted in Figure 4, supporting our claim that the Pt atoms formed a complete monolayer shell on each Au nanosphere. In addition, the lattice mismatch between the Pt shell and Au core was measured to be 4.2%, nearly equal to the theoretical value of 4.0%. Taken together, these data consistency implied that the shell was consisted of pure Pt instead of a Pt–Au alloy.

Using the standard protocol, we were also able to deposit a Pt monolayer shell on the 21 nm Au spheres. Figure S9A shows the particles after Pt deposition. Again, the spherical shape of the particles was preserved due to the involvement of an ultrathin Pt shell. Subsequently, we obtained the atomic ratio of Pt to Au of the 21 nm Au@Pt spheres using ICP-MS (Table 1). The Pt shell thickness was calculated to be ca. 1.06 atomic layer, similar to the value obtained for the 12 nm spheres. These results suggested that a monolayer shell made of Pt could be formed on Au nanospheres with different sizes. To further characterize the Pt monolayer, we obtained a HAADF-STEM image of a 21 nm Au@Pt_1L_ sphere (Figure S9B) and measured the interatomic distances at multiple sites on the same particle (Table S6). As shown in Figure S9C, the first layer along the [111] direction displayed a distance of 1.17 nm across 5 atoms, while the second layer showed a spacing of 1.24 nm. This trend was consistent with the measurement along the [100] direction where the first layer had an interatomic distance of 1.13 nm across 4 atoms and the second layer exhibited 1.17 nm (Figure S9D). Overall, the measurements of interatomic distances were in line with those obtained from 12 nm Au@Pt_1L_ sphere, confirming the presence of a Pt monolayer on the 21 nm Au@Pt_1L_ spheres.

The formation of an ultrathin Pt shell was imperative to maintain the spherical shape taken by the particles. This condition, in turn, critically depended on a balance between the deposition and surface diffusion rates of the freshly deposited Pt atoms. Using the standard protocol, we also increased the concentration of PtCl_4_^2–^ and hence the reduction/deposition rate and the number of Pt atoms deposited on each particle. Specifically, the concentration of PtCl_4_^2–^ was increased by twofold and fourfold for the synthesis of particles shown in Figure S10A,B, respectively. In the case of twofold increase, some particles were deviated from the spherical shape, generating large flat facets that were possibly {111} facets (marked by the red arrow). Further doubling the concentration resulted in higher abundance of irregular particles while a few nanospheres could still be identified (Figure S10B). The deviation from the original spherical shape could be ascribed to the clustering of the freshly deposited Pt atoms into islands on the surface of Au nanospheres (Figure S10C). As the concentration of PtCl_4_^2–^ was increased, the deposition of Pt atoms would switch from a layer-by-layer to an island growth mode owing to the faster reduction kinetics at a higher precursor concentration while the surface diffusion rate of the adatoms remained essentially the same.^60^ The facets exposed on the island should be dominated by {111} because they had the highest atomic coordination number and were most stable relative to {100}, {110}, and high-index facets. As a result, the final products evolved into non-spherical shapes.

Due to their large facets, it was challenging to deposit a complete monolayer of Pt on the surface of both octahedral and cubic nanocrystals under the same condition as in the case of spherical seeds. As shown by ICP-MS analysis, only 0.58 and 0.77 atomic layers of Pt atoms were deposited on octahedral (Figure S11A) and cubic (Figure S11B) seeds, respectively, after 10 min into the synthesis at 27 °C (Table 1), despite that the amount of PtCl_4_^2–^ added was adequate for the formation of one monolayer. We then characterized the samples using HAADF-STEM. As shown in Figure S12A,B, a small monolayer raft was observed on the smooth {100} facets of a Au@Pt_0.77L_ nanocube. The interatomic distance across 4 atoms on this raft was measured to be 0.78 nm (red trace in Figure S12C), which was smaller than the 0.82 nm spacing of the underlying Au (green trace). We also identified a similar raft on the Au@Pt_0.58L_ nano-octahedron (Figure S12D,E). The spacing across 5 atoms on the raft was 1.20 nm (red trace in Figure S12F), smaller than the 1.24 nm of the Au core (green trace). Altogether, the interatomic distance data confirmed the formation of Pt sub-monolayer, rather than a complete monolayer, on both cubic and octahedral Au nanocrystals. The results from ICP-MS and HAADF-STEM implied that, within the same time frame, a more complete Pt monolayer shell could be deposited on the nanospheres compared to their octahedral and cubic counterparts.

The difference in Pt deposition among nanocrystals with varying shapes can be related to the reduction mechanism of PtCl_4_^2–^ precursor on the Au seeds. In the current study, the reduction of PtCl_4_^2–^ undertook a surface reduction mechanism because of the low temperature involved. Typically, the precursor ions adsorbed onto the nucleation sites of the seeds, followed by their reduction to atoms. Since the high-index facets on a spherical seed featured lower packing densities of atoms and thus higher surface energies than the {111} and {100} facets on octahedral and cubic seeds, respectively, they were more active sites for precursor reduction. Accordingly, Pt atoms were deposited more rapidly on spherical seeds, resulting in the formation of a fuller monolayer within the same period of time.

As illustrated in Figure 5, the increased number of nucleation sites available on the surface of spherical seeds also facilitated the adsorption of more precursor ions, resulting in the quicker formation of a monolayer. Specifically, the Pt atoms preferred to nucleate and grow from the low-coordination corners of octahedral and cubic seeds. Each octahedral seed provided six corners for nucleation (the left route), whereas each cubic seed offered eight (the middle route). In contrast, the higher abundance of high-index facets on the surface of a spherical seed allowed many more sites for nucleation (the right route). One might argue that increasing the reaction temperature could also facilitate the reduction of PtCl_4_^2–^ and thus accelerate the deposition of Pt atoms for the creation of a monolayer. However, raising the temperature would promote undesired inter-diffusion between the Pt atoms in the shell and the Au atoms in the core, making it difficult to retain Pt atoms on the outermost layer. Altogether, these results indicated that Au nanospheres were better seeds than their octahedral and cubic counterparts for the conformal deposition of a Pt monolayer shell.

**Figure 5:**
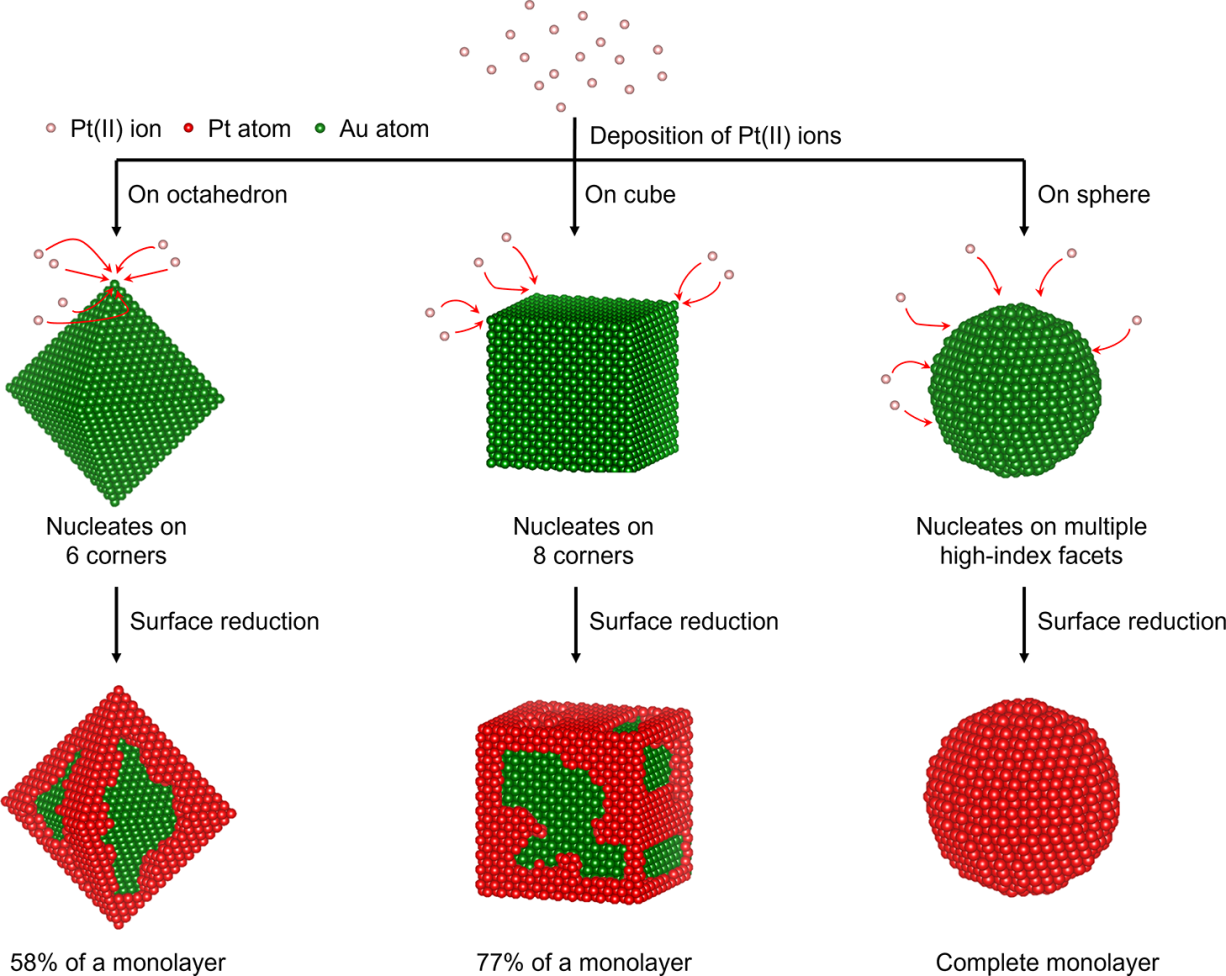
Schematic showing the preferential sites for the nucleation and deposition of Pt atoms while illustrating the role of kinetics in promoting the formation of a Pt monolayer.

The results from our computational study also confirmed that the high-index facets play a crucial role in retaining the Pt atoms on the surface of an Au nanosphere. Table 2 summarizes the differences in energy derived from first principles DFT calculations when a Pt atom was placed on the surface versus in the subsurface of Au (Δ*E*_surf–subsurf_) for a variety of facets. This energy difference is defined as

$$\Delta E_{surf-subsurf}=E_{surface\text{\_}Pt}-E_{subsurface\text{\_}Pt}$$

where $E_{surface\text{\_}Pt}$ is the energy of the most stable configuration in which a Pt atom substitutes an Au atom in the topmost layer of the Au surface model, and $E_{subsurface\text{\_}Pt}$ is the energy of the most stable configuration where a Pt atom substitutes an Au atom in the second topmost layer (subsurface) of the Au surface model. A more detailed description of the computational study can be found in Supporting Information and Figure S13. The results indicated that the Pt atom had a propensity to diffuse from surface into the bulk of the Au domain on all the facets, as indicated by the positive values of Δ*E*_surf–subsurf_ for all facets. However, the tendency for inter-diffusional exchange was significantly mitigated on the high-index facets. Specifically, the values of Δ*E*_surf–subsurf_ for {100} and {111} facets were 0.38 and 0.24 eV per Pt atom, respectively, whereas the values dropped to 0.18, 0.21, and 0.02 eV for {211}, {311}, and {331} facets, respectively. Assuming that the kinetics of the Pt atom diffusion into Au followed the trends determined in the thermochemistry of the process,^29,61–63^ we suggested that the decrease in the calculated energy difference indicated that the Pt atoms on the high-index Au facets would less readily diffuse into the Au host metal, helping preserve the discrete Pt atoms on the surface.

**Table 2:** A Summary of DFT Calculation Results, Showing the Difference in Energy for a Pt Atom When It is Located on the Surface (*E*_surf_) and in the Subsurface (*E*_subsurf_) for the Various Types of Facets on an Au Nanosphere^a^

planes	Δ*E*_surf–subsurf_ (eV)
(100)	0.38
(111)	0.24
(211)	0.18
(311)	0.21
(331)	0.02

a Δ*E*_surf–subsurf_ is the difference in energy when a Pt atom was placed in the surface layer versus in the subsurface layer of the respective Au model slab. Positive entries indicate preference for the subsurface site. A smaller positive value of Δ*E*_surf–subsurf_ indicates a mitigated tendency for the Pt atom to exchange with the underlying Au atoms.

### Catalytic Performance toward the 2-Electron Oxygen Reduction Reaction

The Pt atoms on high-index facets were supposed to have lower coordination numbers than those situated on low-index facets. As such, they could alter the reaction pathway and hence affect the selectivity of a catalytic reaction.^64–69^ To investigate this effect, we evaluated the performance of Au@Pt_1L_ nanospheres as a catalyst for the electrochemical synthesis of H_2_O_2_ via the two-electron ORR. We chose ORR as a model reaction because it could produce two different products depending on the catalytic surface and thus the reaction pathway involved. Notably, it was documented in the literature that the short Pt–Pt distances associated with the closely-packed Pt atoms on low-index facets favored scission of the O–O bond in O_2_ molecules, facilitating the formation of H_2_O through the four-electron pathway. In contrast, the elongated Pt–Pt bond associated with the less densely packed Pt atoms on high-index facets could suppress the breaking of O–O bond, promoting the formation of H_2_O_2_ via the two-electron pathway.^18^

We benchmarked the performance of the Au@Pt_1L_ nanospheres in the electrochemical formation of H_2_O_2_ against Au nanospheres, as well as commercial Pt/C that was largely covered by {111} and {100} facets on the surface.^70^ As shown in Figure 6A,B, the Au@Pt_1L_ nanospheres (the green trace) showed nearly fourfold increase in terms of disk current, which corresponded to a higher overall ORR activity when compared to the Au nanospheres (the red trace). The improvement in activity toward ORR again validated the presence of active Pt atoms on the surface of the Au@Pt_1L_ nanospheres. Besides, the selectivity of the Au@Pt_1L_ nanospheres toward H_2_O_2_ was ca. 20% at 0.35 V_RHE_ (detailed calculations are provided in the Supporting Information), which was greater than that (0%) of Pt/C. The improvement in selectivity suggested that the Pt atoms on the high-index facets indeed favored the production of H_2_O_2_ without breaking the O–O bond in O_2_ molecules. For the same reason, the Au@Pt_1L_ nanospheres also featured a higher ring current, which corresponded to a higher activity toward H_2_O_2_, than the Pt/C. Additionally, the onset potential of the Au@Pt_1L_ nanospheres was negatively shifted by ca. 0.2 V_RHE_ relative to that of the Pt/C, indicating that the Au@Pt_1L_ nanospheres effectively suppressed the four-electron pathway responsible for the production of H_2_O. Altogether, the electrochemical data confirmed that the Pt atoms on the high-index facets favored the two-electron pathway to produce H_2_O_2_ as the final product during ORR.

**Figure 6:**
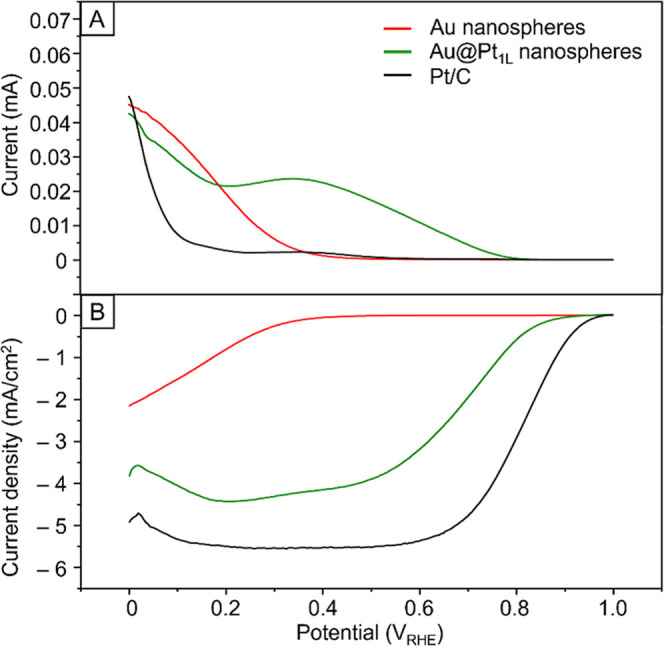
(A) Ring current versus disk potential, showing the activity toward H_2_O_2_ for the Au nanospheres, Au@Pt_1L_ nanospheres, and Pt/C. (B) Disk current versus disk potential, comparing the overall activity toward ORR.

Apparently, the selectivity of the Au@Pt_1L_ nanospheres toward H_2_O_2_ was too low to be considered for practical use. We were able to address this issue by depositing a monolayer comprised of a Pt–Au alloy rather than pure Pt. Following the standard protocol, we injected a pre-mixed solution of aqueous K_2_PtCl_4_ and HAuCl_4_ into the growth solution at different feeding ratios while the total concentration of the metal precursors (i.e., PtCl_4_^2–^ plus AuCl_4_^–^) was fixed at the same level as in the standard protocol to ensure that only a monolayer of metal atoms would be deposited. The products are collectively termed as Au@PtAu hereafter. Figure 7 shows TEM images taken from the samples obtained at different feeding ratios of PtCl_4_^2–^ to AuCl_4_^–^. Since only a monolayer made of a Pt–Au alloy was deposited, all samples displayed a uniform diameter of 12 nm, as well as the preservation of spherical shape, suggesting the retention of the high-index facets.

**Figure 7:**
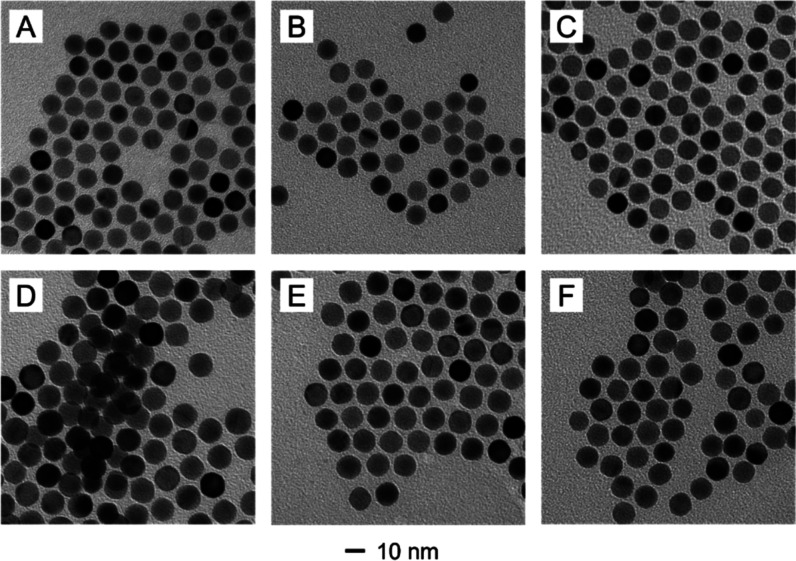
TEM images taken from the samples prepared by depositing a monolayer made of a Pt–Au alloy on the surface of 12 nm Au spheres at different feeding ratio of Pt­(II) to Au­(III): (A) 5:95; (B) 10:90; (C) 15:85; (D) 25:75; (E) 50:50; (F) 75:25.

Figure 8A shows the catalytic selectivity toward H_2_O_2_ for Au@PtAu nanospheres with different feeding ratios of PtCl_4_^2–^ to AuCl_4_^–^. The catalytic performance was indeed improved by switching from pure Pt to Pt–Au alloys for the monolayer coating. As shown in Figure 8B, the selectivity of different samples at 0.35 V_RHE_ increased from 20% to the highest value of 80% as the feeding ratio of Pt­(II) to Au­(III) decreased from 100:0 to 15:85, which was comparable to several recently reported catalysts toward two-electron ORR.^71–73^ This improvement in selectivity could be attributed to two reasons. Firstly, the Au atoms in the Pt–Au alloys geometrically separated the Pt atoms from each other, lengthening the average Pt–Pt distance. As discussed earlier, such a configuration favored the electrochemical synthesis of H_2_O_2_ by preventing the scission of O–O bond. Secondly, Au, which exhibited a higher electronegativity than Pt, could pull electron density away from neighboring Pt atoms. Such an electronic interaction weakened π-back donation from Pt to oxygen, helping preserve the O–O bond. Taken together, alloying Pt with Au avoided the O–O bond from breaking during ORR, resulting in a higher selectivity toward H_2_O_2_. Besides, Figure S14 shows a gradual negative shift of the onset potential as the feeding ratio of PtCl_4_^2–^ to AuCl_4_^–^ decreased from 100:0 to 15:85. This shift indicated that the Au@PtAu nanospheres had greater capability to suppress the four-electron pathway at a lower feeding ratio of PtCl_4_^2–^ to AuCl_4_^–^, further validating the role of Au in modulating the catalytic performance of neighboring Pt atoms. It should be noted that the overall ORR activity decreased with the feeding ratio of PtCl_4_^2–^ to AuCl_4_^–^, which could be ascribed to the reduced exposure of Pt atoms on the surface of the particles at lower feeding ratios.

**Figure 8:**
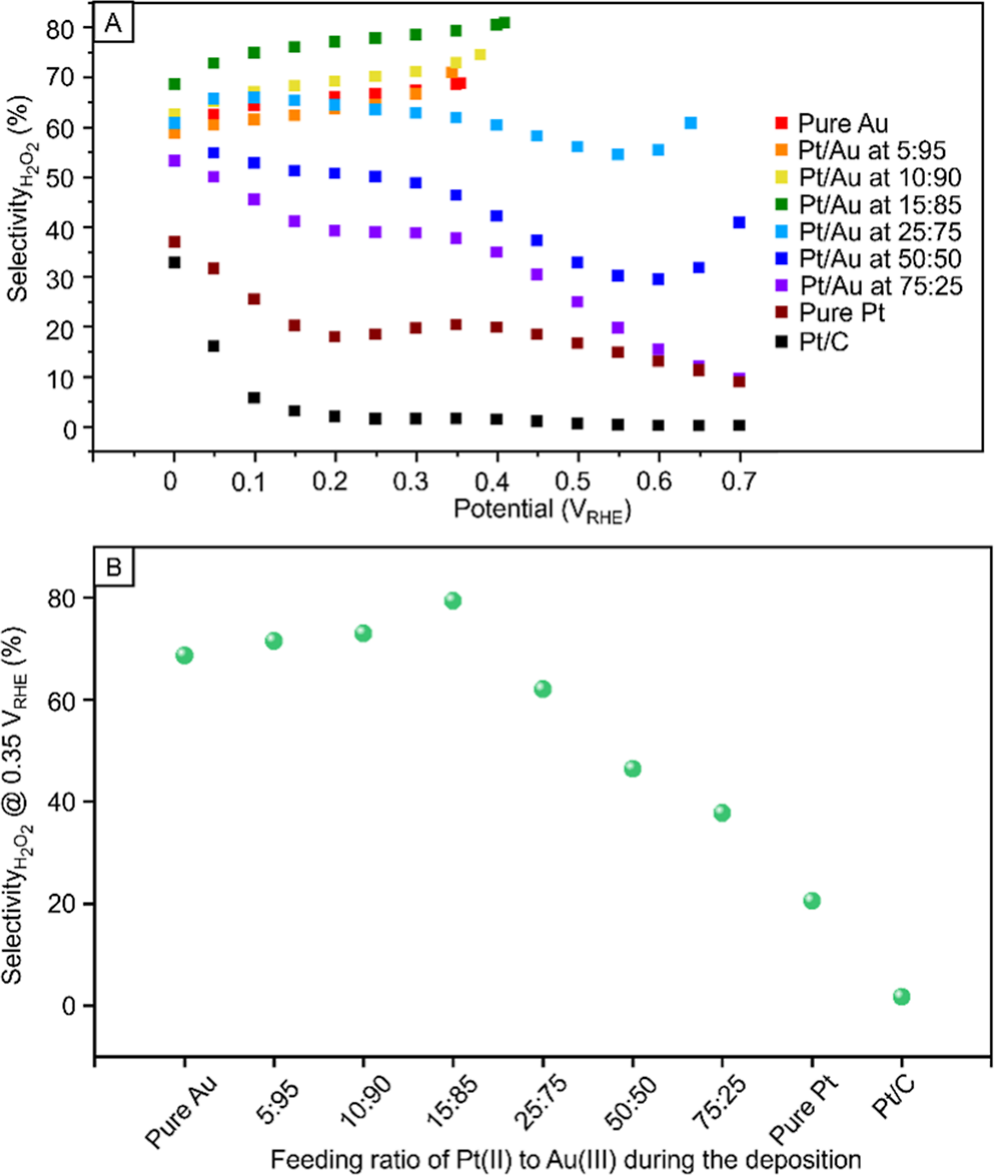
(A) Selectivity toward H_2_O_2_ versus disk potential for the 12 nm Au@PtAu spheres synthesized at different feeding ratios of PtCl_4_^2–^ to AuCl_4_^–^. (B) Selectivity toward H_2_O_2_ at 0.35 V_RHE_ for the different samples of nanospheres.

The selectivity of the Au@PtAu nanospheres toward H_2_O_2_ started to drop when the feeding ratio of PtCl_4_^2–^ to AuCl_4_^–^ was further increased and it eventually decreased to 70% at 5:95. Additionally, we recorded a decrease (ca. 30%) in the ring current when the feeding ratio of PtCl_4_^2–^ to AuCl_4_^–^ decreased from 15:85 to 5:95. The reduction in both selectivity and activity implied that the inadequate supply of Pt atoms for the 5:95 sample resulted in a very low density of Pt atoms on the surface, making the particles more inert toward ORR. Altogether, the selectivity of different samples exhibited a volcano-type dependence on the feeding ratio between the two precursors. This result indicated that alloying Pt with Au could fine-tune the catalytic properties of Pt. When the feeding ratio of PtCl_4_^2–^ to AuCl_4_^–^ was set to 15:85, we achieved the highest selectivity.

For the catalytic work, we focused on the deposition of a monolayer made of pure Pt or Pt–Au alloy on the Au spheres with diameter of 12 nm. As the feeding ratio of Pt­(II) to Au­(III) decreased from 100:0 to 15:85, the selectivity toward two-electron ORR increased markedly, highlighting the potential of the Au@PtAu nanospheres as a catalyst for electrochemical production of H_2_O_2_. However, due to the similar atomic sizes of Pt and Au, the core–shell structure might be prone to alloying under the electrochemical conditions, which tended to compromise stability. We took HAADF-STEM image of the Au@Pt_1L_ nanospheres after CV cycling for 20 rounds between 0.05 and 1.0 V_RHE_. As shown in Figure S15A, the spherical shape of the particle was well preserved. However, the EDX line-scan (Figure S15B) indicated that the Pt signal was concentrated in the core rather than uniformly distributed across the particle as in Figure 3F. This result indicated that the Pt atoms in the shell started to alloy with the Au atoms in the core. Moving forward, it is essential to ensure the durability of the catalyst for practical applications. In parallel, it is also important to understand how the dilution of Pt atoms by Au influences the reaction pathway during ORR.^74^ In particular, the involvement of Au, which is a plasmonic metal, provides an opportunity to use in situ surface-enhanced Raman scattering to probe the adsorbate-metal interactions during the electrochemical reaction, therefore, offering mechanistic insight into H_2_O_2_ formation.^75^ In addition, recent publications also demonstrated that in situ electrochemical 4D-STEM and X-ray spectroscopy could serve as a tool for mechanistic study.^76,77^

## Conclusion

In summary, we have demonstrated the advantages of Au nanospheres over octahedral and cubic counterparts for the synthesis of nanocatalysts with a Pt shell of only one atomic layer in thickness. Specifically, the abundance of small high-index facets on the surface of nanospheres makes it easy for the Pt atoms to nucleate and then spread into a monolayer through surface diffusion. In support through our trained Allegro MLIP, we show that the surface of the Au nanospheres exhibit a large fraction of CN ≤ 7 that are relatively uniformly distributed over the surface. Additionally, the high-index facets help retain the freshly deposited Pt atoms on the outermost surface by suppressing their inter-diffusion exchange with the underlying Au atoms. When switching to a monolayer comprised of a Pt–Au alloy, we can optimize both the activity and selectivity toward the two-electron ORR, facilitating the electrochemical production of H_2_O_2_. This method should be extendible to the preparation of other core–shell nanocatalysts intended for additional reactions.

## Supplementary Material



## Data Availability

The MLIP training and validation data generated in this work are available at https://github.com/MarvikakisGroup/au-nanospheres-mlip-data.

## Notes

Experimental section, TEM images of Au seeds and octahedral and cubic Au@Pt nanoparticles, assignment of the high-index facets, additional electrochemical data, additional computation details (PDF)
