# Supplementary material for: What Makes Au Nanospheres Superior to Octahedral and Cubic Counterparts for the Deposition of a Pt Monolayer Shell?
Source: J Am Chem Soc. 2025 Jul 10;147(29):25273–86. doi: 10.1021/jacs.5c03700 (PMC12291435; doi:10.1021/jacs.5c03700)
Supplement: Supplementary file 1 [file ja5c03700_si_001.pdf]

Supporting Information

What Makes Au Nanospheres Superior to Octahedral and Cubic Counterparts for the Deposition of a Pt Monolayer Shell?

Kei Kwan Li,[†1] Lance Kavalsky,[‡1] Marc Figueras-Valls,[‡] Yong Ding,[§] Manos Mavrikakis,[‡,*] and Younan Xia[†,¶,*]

[1]These author contributed equally.

[†]School of Chemistry and Biochemistry, Georgia Institute of Technology, Atlanta, Georgia 30332, United States

[‡]Department of Chemical and Biological Engineering, University of Wisconsin–Madison, Madison, Wisconsin, 53706, United States

[§]School of Materials Science and Engineering, Georgia Institute of Technology, Atlanta, Georgia 30332, United States

[¶]The Wallace H. Coulter Department of Biomedical Engineering, Georgia Institute of Technology and Emory University, Atlanta, Georgia 30332, United States

*Corresponding authors. E-mails: emavrikakis@wisc.edu (for the computational study) and younan.xia@bme.gatech.edu (for the experimental study)

EXPERIMENTAL SECTION

Chemicals and Materials. Gold(III) chloride trihydrate ($HAuCl_4 \cdot 3H_2O$, ≥99.9%), potassium tetrachloroplatinate(II) (K_2PtCl_4, ≥99.9%), sodium borohydride ($NaBH_4$, 98%), ascorbic acid (AA, ≥99.0%), cetyltrimethylammonium bromide (CTAB, ≥99.0%), cetyltrimethylammonium chloride (CTAC, 25% in water), and isopropanol were all obtained from Sigma-Aldrich and used as received. Deionized water with a resistivity of 18.2 MΩ·cm at room temperature was used throughout the experiments.

Synthesis of the 12-nm Au Spheres Capped by CTAB/C. We followed a published protocol to synthesize CTAC/B-capped Au nanospheres of 12 nm in diameter. In brief, Au clusters were firstly prepared by mixing 5 mL of aqueous CTAB (200 mM) and 5 mL of aqueous $HAuCl_4$ (0.5 mM) in a 20-mL glass vial, followed by the introduction of freshly-prepared 0.6 mL of aqueous $NaBH_4$ (10 mM) in one shot at room temperature. The mixture was placed on an orbital shaker at a speed of 270 rpm for 2 min and then kept undisturbed for at least 3 h at 27 °C to allow the $NaBH_4$ to completely decompose. Meanwhile, 2 mL of aqueous CTAC (200 mM) and 2 mL of aqueous $HAuCl_4$ (0.5 mM) were mixed in a separate 20-mL glass vial, followed by the one-shot injection of 1.5 mL of aqueous AA (100 mM) under magnetic stirring at a speed of 600 rpm and at 27 °C. Then, 50 µL of the suspension of Au clusters was introduced. The reaction was allowed to proceed at 27 °C for 15 min. The solid products were collected by centrifugation at 14500 rpm for 30 min and washed once with water. After washing with water and removal of supernatant, the particles were re-dispersed in 1 mL of aqueous CTAC (20 mM). The concentration of Au in this suspension was determined to be 0.8 mM by ICP-MS analysis.

Synthesis of the 21-nm Au Spheres Capped by CTB/C. In a standard protocol, a mixture of 2 mL of aqueous CTAC (100 mM), 130 µL of aqueous AA (10 mM), and 100 µL of the 12-nm Au spheres was mixed in a 20-mL glass vial under magnetic stirring at a speed of 600 rpm and at 27 °C. Then, 2 mL of aqueous $HAuCl_4$ (0.5 mM) was introduced dropwise using a syringe pump at an injection rate of 2 mL/h. Afterwards, the reaction was allowed to proceed for another 10 min at 27 °C and the solid products were collected by centrifugation at 14500 rpm for 30 min and washed once with water. The particles were re-dispersed in 20 mM aqueous CTAC after washing and extraction of supernatant.

Synthesis of the 20-nm Au Octahedra Capped by CTAB/C. In a standard protocol, DMF (5.8 mL) containing 0.162 mM of $HAuCl_4$ and 0.213 M of PVP (M.W. = 55K) was placed in a 20-mL

glass vial at 27 °C, followed by the addition of a 12-nm Au spheres (0.1 mL) and water (0.1 mL). The vial was then heated in an oil bath at 80 °C under magnetic stirring at a speed of 600 rpm for 1 h. After that, the solid product was collected by centrifugation at 14500 rpm for 10 min and then re-dispersed in 1 mL of aqueous CTAC (20 mM). Subsequently, 1 mL of octahedral suspension was mixed with 5 mL of aqueous CTAC and 650 μL of aqueous AA (10 mM) in a 20-mL glass vial under magnetic stirring at a speed of 600 rpm and at 27 °C. Then, 500 μL of aqueous $HAuCl_4$ solution (0.25 mM) was added in one-shot at the same stirring speed and temperature. The reaction was then allowed to proceed for another 10 min at 27 °C. The solid products were collected by centrifugation at 14500 rpm for 10 min and then re-dispersed in 20 mM aqueous CTAC after extraction of supernatant.

Synthesis of the 22-nm Au Cubes Capped by CTAB/C. In a standard protocol, 2 mL of aqueous CTAC (100 mM) was mixed with 130 μL of aqueous AA (10 mM), 10 μL of aqueous KBr (125 mM), and 100 μL of the 12-nm Au spheres in a 20-mL glass vial under magnetic stirring at a speed of 600 rpm and at 27 °C, followed by the one-shot injection of 2 mL of aqueous $HAuCl_4$ (0.5 mM). Then, the reaction was allowed to proceed for 25 min at 27 °C. The resultant nanocubes were collected by centrifugation at 14500 rpm for 10 min, washed with water one more time at the same speed but for 5 min, and then re-dispersed into 20 mM aqueous CTAC after washing and extraction of supernatant.

FTIR measurement. All Au nanocrystals were collected again by centrifugation at 14500 rpm for 30 min and then washed with water one more time. The solids were re-dispersed in water after washing and extraction of supernatant for FTIR measurement to exclude the effect brought by CTAC solution.

Synthesis of the 12-nm Au@Pt_{1L} Spheres. In a standard protocol, 0.2 mL of the suspension of 12-nm Au spheres was mixed with 2 mL of aqueous CTAC (100 mM) and 0.13 mL of aqueous AA (10 mM) in a 20-mL glass vial under magnetic stirring at a speed of 600 rpm and at 27 °C. Then, 25 μL of aqueous K_2PtCl_4 (0.5 mM) was introduced in one shot. Afterwards, the reaction was allowed to proceed at 27 °C for 10 min. The solid products were collected by centrifugation at 14500 rpm for 30 min and then re-dispersed in water.

For depositing a monolayer of Pt on Au octahedral and cubic nanocrystals to synthesize Au@Pt_{1L} octahedra and cubes, 0.2 mL of the suspension of Au octahedral, cubic, and larger spherical seeds (concentration of Au = 0.8 mM, 1.0 mM, 1.6 mM in 20 mM aqueous CTAC,

respectively) were used. Everything else was kept the same as that for the Au nanospheres.

For the synthesis of 12-nm Au@$PtAu_{1L}$ spheres at different feeding ratios of Pt(II) to Au(III), a mixture of aqueous K_2PtCl_4 and $HAuCl_4$ at different molar ratios was used (with a total concentration of metal ions being 0.5 mM, see main text for details). Everything else was kept the same as that for the Au nanospheres.

Characterizations. Transmission electron microscopy (TEM) images were taken using Hitachi HT7700 microscope operated at 120 kV. High-angle annular dark-field (HAADF) scanning TEM (STEM) image and energy-dispersive X-ray spectroscopy (EDX) mapping and line-scanning were conducted using an aberration-corrected Hitachi HD-2700 200 kV STEM equipped with a Brüker Quantax 400/S–STEM EDX detector. The metal contents of the samples were analyzed using an inductively-coupled plasma mass spectrometer (ICP-MS, NexION 300Q, PerkinElmer). The UV-vis spectra were recorded on a Cary 60 spectrometer (Agilent Technologies, Santa Clara, CA).

Electrode Preparation. The working electrode was polished with 0.3-μm Al_2O_3 slurry and then with 0.05-μm Al_2O_3 slurry. Subsequently, it was washed with water and ethanol. The catalyst ink was prepared by mixing the metal nanoparticles with carbon black (Vulcan XC-72) at a mass ratio of 1:4 in a mixture of water and isopropanol (4:1 by vol.) under ultrasonication in an ice bath for 3 h. The carbon-supported catalyst was collected by centrifugation and re-dispersed in a mixture of water and isopropanol (4:1 by vol.) containing 5 wt.% Nafion. The atomic ratios of Pt to Au in the final inks were provided in Table S7. Afterwards, 9 μL of the suspension was deposited on a precleaned glassy carbon rotating ring-disk electrode (RRDE) (Pine Research Instrumentation) with a geometric area of 0.237 cm^2 and then dried in air. A commercial Pt/C (20 wt.%) from Sigma-Aldrich was used for benchmarking.

Electrochemical Measurements. The measurements were performed in a standard three-electrode system using a CHI 760E potentiostate (CH Instruments). We used a saturated calomel electrode (BASi, pre-calibrated with a reversible hydrogen electrode, RHE) and a Pt mesh as the reference and counter electrodes, respectively. All the potentials were converted to the values in reference to RHE. Before measurements, all solutions were purged and saturated with Ar or O_2. To measure ORR activity, the catalyst was first cycled 40 times between 0.05–1.1 V_{RHE} at 100 mV s^{-1} in Ar-saturated 0.1 M $HClO_4$. A background CV was obtained under the same conditions except that the scan rate was reduced to 10 mV s^{-1}. ORR measurements were conducted by cycling the

potential 20 times between 0–1.0 V_{RHE} at 10 mV s^{-1} in O_2-saturated 0.1 M $HClO_4$ at 1,600 rpm. The collection efficiency for the RRDE was 25.6% and the ring current was kept constant at 1.2 V_{RHE}. All the electrochemical data were *iR*-compensated at 85%. The onset potential was defined as the value at which the first derivative starts increasing.

The selectivity of each catalyst toward H_2O_2 was determined based on the currents of ring and disc electrodes according to the following equation:

$$\text{Selectivity toward } H_2O_2\text{: } H_2O_2\,(\%) = 200 \times \frac{\frac{I_R}{N}}{I_D + \frac{I_R}{N}} \tag{S1}$$

where I_R and I_D corresponded to the ring and disk currents, respectively. The values of different meal nanoparticles were collected and shown in Figure S10. *N* was the collection efficiency (25.6%, as calibrated using $K_3Fe(CN)_6$). A fixed potential of 1.2 V_{RHE} was applied to the ring during the entire testing process so that O_2 would not be reduced while all the radially-dispersed H_2O_2 would be oxidized.

Density Functional Theory calculations. All computational studies were conducted with periodic density functional theory (DFT) employing the Perdew-Burke-Ernzerhof (PBE)[1] exchange-correlation functional within the generalized gradient approach (GGA). Dispersion terms were included using the D3 method of Grimme with zero-damping function.[2] The calculations were carried out using the Vienna Ab initio simulation package (VASP),[3] where the Kohn-Sham equations were solved using a plane-wave basis set with an energy cutoff of 400 eV. The effect of core electrons on the valence regions was included using the projected augmented wave method as implemented by Kresse and Joubert.[4]

The combination of D3 and PBE accurately describes the electronic structure of Au, the major element in the systems studied experimentally, as demonstrated by the calculated cohesive energy (3.68 eV)[5] and bulk lattice constant (4.1 Å)[6] of Au, which closely match their experimental values (3.81 eV and 4.079 Å, respectively). The thermodynamic preference of Pt to exist on the surface or subsurface of Au nanoparticles as a function of the facet type was studied by comparing the total energy of isomer slabs of Au with one Pt atom substituting an Au atom either in the surface or in the subsurface of the respective slabs. The Au facets studied were those observed in experiments, namely Au(111), Au(100), Au(211), Au(311), and Au(331). For facets exhibiting symmetrically inequivalent atoms, all possible Au-Pt substitutions were calculated, and only the most stable configuration was accounted for in the analysis. To account for surface-bulk transitions,

all slab models consisted of 4 atomic layers, where the two bottommost layers were constrained at the bulk atomic coordinates, and the two topmost layers were fully relaxed. The supercell size of the slab models was selected to prevent artificial interactions between Pt atoms in adjacent periodic images, mimicking a dilute Pt substitution in Au. The numerical integration was carried out in the reciprocal space using Monkhorst-Pack[7] grids of 6x6, 4x4, 6x5, 3x3, and 4x3 for the Au(111), Au(100), Au(211), Au(311), and Au(331) facets, respectively. Convergence with respect to calculation parameters was ensured in all cases. Model depictions of each termination and the most stable locations of the surface and subsurface Pt atoms for the relevant substitutions are illustrated in Figure S9.

Generating Machine-Learned Interatomic Potential Training Data. Compiling a varied set of DFT data to train our MLIP on, we selected several input structures exhibiting a range of local gold environments including: (i) bulk gold, (ii) extended terrace and step facets, (iii) adatom clusters and vacancies, (iv) non-stoichiometric overlayers, (v) icosahedron nanoparticles, (vi) cuboctahedron nanoparticles, and (vii) Wulff construction nanoparticles. The included Wulff constructions were generated using the Atomic Simulation Environment[8,9] with surface energies tabulated in the Materials Project database.[10,11] The input structures were supplied to the FLARE algorithm (commit cb57c6b)[12,13] to flesh out an information-rich set of atomic configurations through active learning. For all DFT calls made by FLARE, the same calculator settings as above were used, with all k-point meshes selected via 45/a x 45/b x 1 for all surfaces, 45/a x 45/b x 45/c for bulk, and Gamma-point sampling for nanoparticles where a, b, and c are the unit cell dimensions in Å. Selecting the settings for running the FLARE algorithm itself, we used a timestep of 1 fs, a normalized dot product kernel, a cutoff radius of 6 Å, and the velocity Verlet algorithm for propagating the system through time. All input structures were initially randomly jittered with an amplitude of 0.05 Å to ensure symmetry breaking. For each input structure, 3 independent FLARE runs were conducted at 300 K, 500 K, 1000 K, and 1200 K to encourage varying degrees of energy landscape exploration. Every independent FLARE calculation selected 5 environments at random to initialize the model, and a maximum of 20 environments was allowed to be added to the training set at every subsequent iteration of the algorithm. Hyperparameters of the FLARE gaussian process model were optimized only between the 5th and 20th DFT calls within a single run. Each run was conducted up until a minimum of 1 ps, or until consecutive DFT calls were spaced by at least ~0.2 ps. The final training set was an aggregate of the collected DFT data from

the FLARE runs, resulting in a training set of 1177 Au structures. A breakdown of the dataset is provided in Table S3. All numerical validation tests were generated following an identical procedure with independent runs (Table S4).

Allegro MLIP Training. To explicitly model the full Au nanospheres at DFT-level accuracy, we trained an equivariant Allegro MLIP (commit 00b6038)[14] on the training data described in the previous section. For numerical accuracy, we used the default float64 data type. This model defines local environments via pairwise interactions, and we set the cutoff radius to 6 Å. Prioritizing accuracy in the energy predictions of our potential, we defined the energy loss using mean squared error (MSE) per atom and set its coefficient to be 100 in the loss function. The coefficient for forces in the loss function was selected to be 1. Our model architecture and training hyperparameters were selected based on hyperparameter optimization across validation tests covering bulk and surface properties and informed by recommended default parameters in the Allegro documentation. We used 16 tensor features and a maximum rotation order of 2 with full O(3) symmetry. The multilayer perceptron (MLP) for the two-body embedding had the dimensions of [128, 256, 512, 1024]. In the hidden layers, the latent (MLP) had dimensions of [1024, 1024, 1024] whereas the embedding MLP was a single linear layer. All MLPs in our model, except the hidden layer embedding MLP, used sigmoid linear unit (SiLU) nonlinearities. The final energy MLP had a dimension of [128]. For training, we used an 80%/20% training-validation split with random shuffling and a batch size of 4. We chose an initial learning rate of 0.001 which was reduced on plateau, as implemented in Allegro's ReduceLROnPlateau scheduler, until a rate of 1.0×10^{-5} was reached, stopping training.

Calculating Surface Energies. For calculating the surface energies of extended surfaces, we used the following expression:

$$\sigma = \frac{E_{tot} - N * E_{bulk}}{2A}$$

where E_{tot} is the total energy of the slab consisting of N Au atoms in a 1×1 supercell, E_{bulk} is the bulk energy of Au calculated using the linear fit method[15] applied to the Au(100) surface, and A is the area of the cell in the x-y plane. Similarly, for the nanoparticles the surface energy was calculated using:

$$\sigma = \frac{E_{tot} - N * E_{bulk}}{A}$$

where A is the surface area estimated using the alpha-shape method[16] as implemented in Ovito.[17]

The default settings of a probe sphere radius of 4 and smoothing level of 8 was applied to calculate the surface areas.

Simulating the Au Nanospheres. Molecular dynamics calculations were conducted using the LAMMPS software package,[18,19] which was patched with pair allegro to support Allegro force fields.[20] Non-periodic boundary conditions and a timestep of 2 fs was used in the NVT canonical ensemble for all calculations. Temperature was controlled via the Nose-Hoover thermostat with a target of 300 K. The system was thermally equilibrated over 120 ps, followed by an additional 60 ps of production simulation which we used for our analysis. To analyze the fraction of the nanosphere surface possessing different coordination numbers, coordination analysis is conducted with a cutoff radius of 3.5 Å and surface atoms are identified via the alpha-shape method,[16] with both methods applying the Ovito implementation.[17] The thermodynamic and structural information of the nanosphere was recorded every 0.2 ps and used for averaging properties.

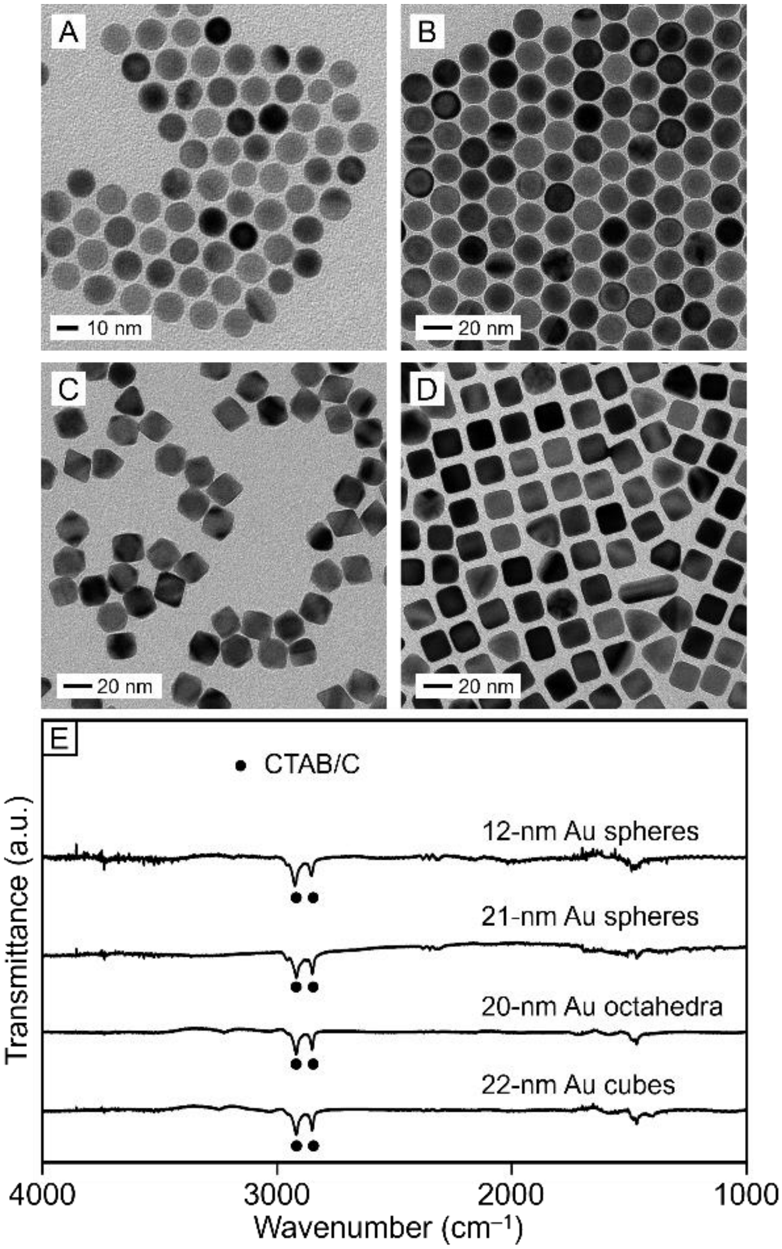

Figure S1. Typical TEM images of the (A) 12-nm Au spheres; (B) 21-nm Au spheres; (C) 20-nm Au octahedra; and (C) 22-nm Au cubes to be explored as seeds for Pt deposition. (D) FTIR spectra recorded from the Au nanocrystals with different shapes.

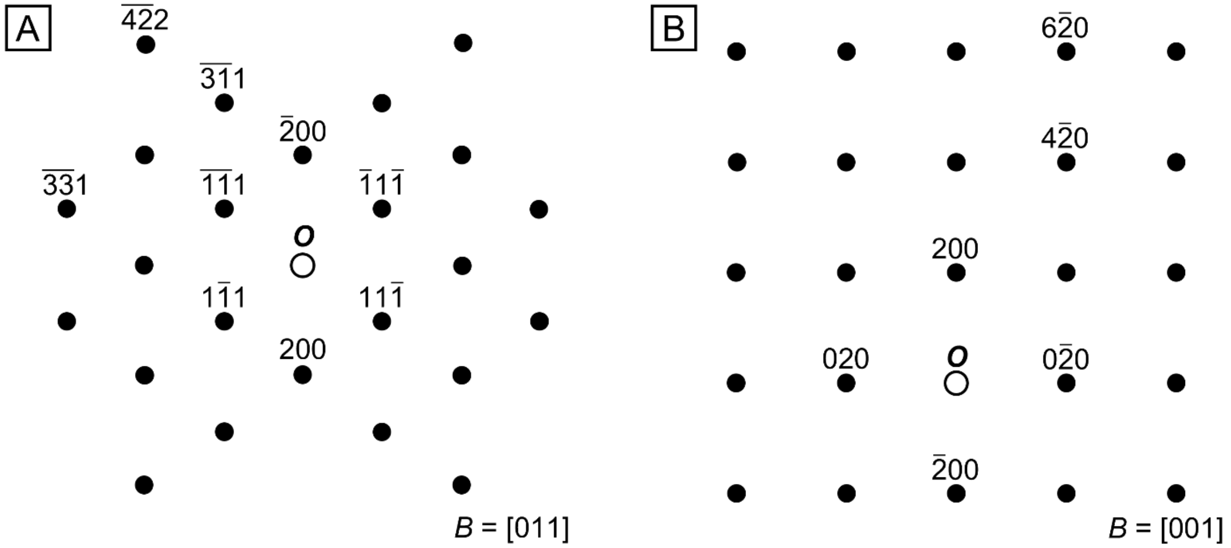

Figure S2. Theoretical FFT patterns of a face-centered cubic (*fcc*) metal along (A) [011] and (B) [001] directions, respectively.

Table S1. (A) Theoretical angles between the high-index planes and {111} or {200} planes, as obtained from Figure S2A. (B) The angles measured between the facets formed by the lines and the {111}/{100} facets in Figure 1A. (C) Assignment of the facets exposed on the surface of the 12-nm Au sphere shown in Figure 1A.

(A)

{hkl}	Theoretical angle between the high-index plane and {111}*	{hkl}	Theoretical angle between the high-index plane and {200}*
211	158.5 °	311	154.5 °
331	157.5 °		

* The values were obtained by measuring the angles between the spots of hkl and the spots of 111/200 in Figure S2A.

(B)

Lines	Measured angle between the facets formed by the lines in Figure 1A and (111)	Lines	Measured angle between the facets formed by the lines in Figure 1A and (100)
Purple	158.6 °	Orange	154.4 °
Blue	157.6 °		

(C)

Lines	Corresponding high-index facets {hkl}*
Purple	211
Orange	311
Blue	331

*The results were obtained by matching the angles in Table S1A with those in Table S1B.

Table S2. (A) Theoretical angles between the high-index planes and {200} planes, as obtained from Figure S2B. (B) The angles measured between the facets formed by the lines and the {111}/{100} facets in Figure 1B. (C) Assignment of the facets exposed on the surface of the 12-nm Au sphere shown in Figure 1B.

(A)

{hkl}	Theoretical angle between the high-index planes and {200}*
210	153.4 °
310	161.6 °

* The values were obtained by measuring the angles between the spots of hkl and the spots of 200 in Figure S2B.

(B)

Lines	Measured angle between the facets formed by the lines in Figure 1B and (100)
Yellow	154.8 °
Blue	162.5 °

(C)

Line	Corresponding high-index facets {hkl}*
Yellow	210
Blue	310

*The results were obtained by matching the angles in Table S2A with those in Table S2B.

Table S3. MLIP Training set summary. The number of DFT calculations for each system is an accumulation of all FLARE runs using that input structure (excluding the FLARE runs used to generate the internal validation tests). Systems labelled Au_x/Au_y ($x \neq y$) are non-stoichiometric overlayers with x atoms in the overlayer interfaced with y atoms in the supporting underlayer. A total of 1177 DFT calculations were used for the entire training set.

System	# of DFT Calculations	System	# of DFT Calculations
Au_{bulk}	24	Au_{13}/Au_{16}	32
Au(111)	53	Au_{16}/Au_{13}	40
Au(211)	37	Au_{13}/Au_{9}	47
Au(874)	71	Au_{9}/Au_{13}	35
Au(100)	42	Au_{9}/Au_{7}	55
Au(310)	58	Au_{7}/Au_{9}	45
Au(311)	54	Au(100)+hexagonal overlayer	25
Au(210)	56	Au(100)+Adsorbate Row Reconstruction	31
Au(331)	61	Au_{147}^{ico}	46
Au(111)+adatom+vacancy	46	Au_{147}^{cubo}	40
Au(111)+dimer+2 vacancies	64	Au_{153}^{Wulff}	49
Au(111)+trimer+3 vacancies	33	Au_{327}^{Wulff}	36
Au(111)+tetramer+4 vacancies	28		
Au(111)+pyramid cluster	69		

Table S4. MLIP Validation set summary. The two columns on the left correspond to the internal validation tests, and the two columns on the right correspond to the external validation tests. All configurations were generated using FLARE following an identical procedure as for generating the training data. A total of 658 DFT calculations were used for the entire validation set.

System	# of DFT Calculations	System	# of DFT Calculations
Au_{bulk}	24	Au_{309}^{ico}	41
Au(100)	41	Au_{309}^{cubo}	40
Au(111)	41	Au_{55}^{ico}	63
Au(210)	56	Au_{55}^{cubo}	48
Au(211)	38	Au(332)	47
Au(310)	63	Au(511)	47
Au(311)	55		
Au(331)	54		

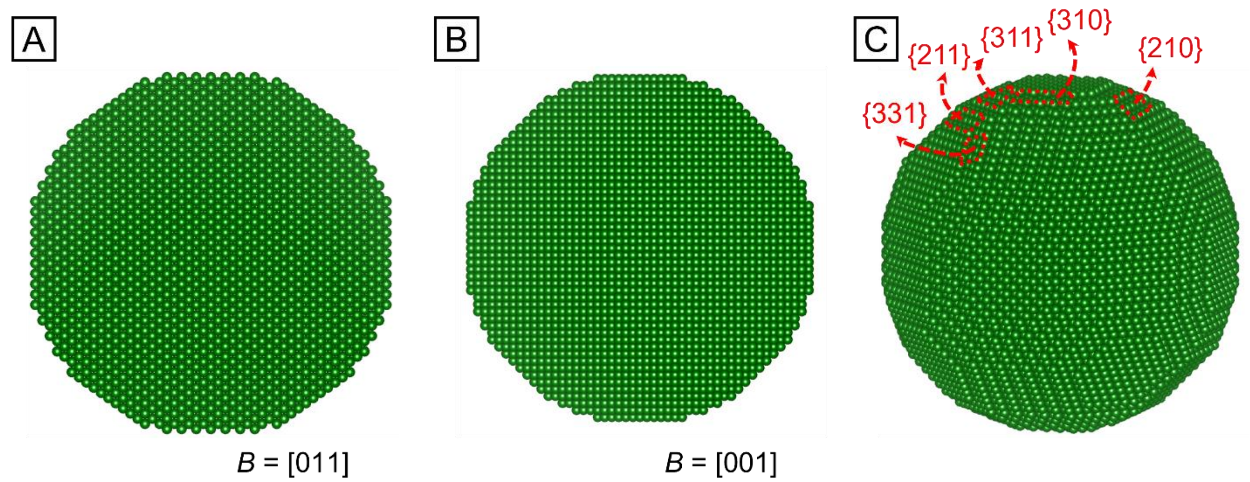

Figure S3. Atomic models of a nanosphere viewed along (E) [011], (F) [001], and (G) arbitrary directions, respectively.

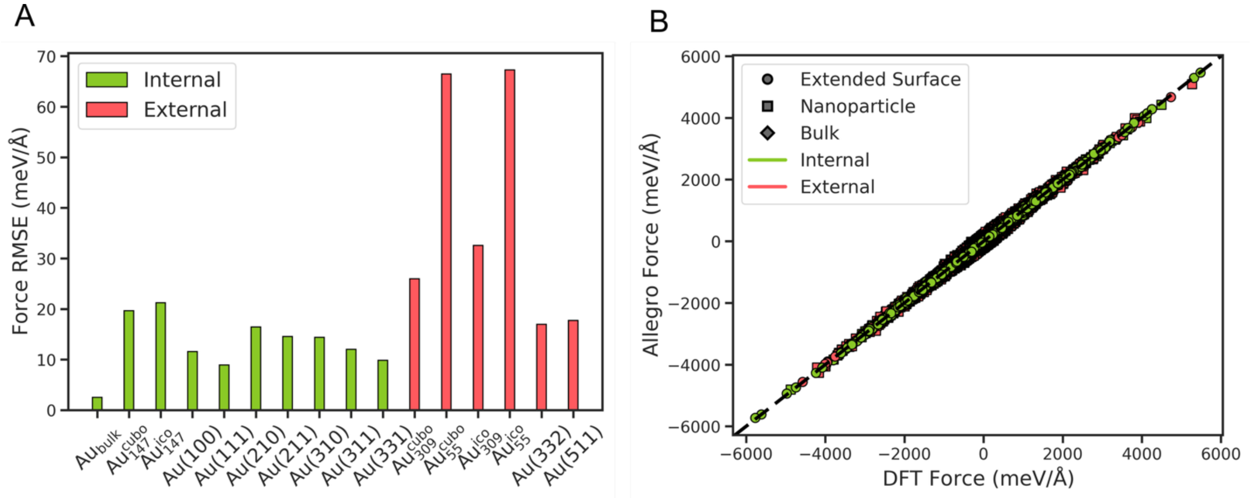

Figure S4. (A) Calculated root-mean squared error (RMSE) for internal and external validation tests. Internal (external) validation tests refer to configurations collected by FLARE where their input structure is (not) used in constructing the training set. (B) Parity plot of DFT calculated forces and corresponding predictions from our Allegro MLIP. A perfect surrogate has all points lying along the diagonal.

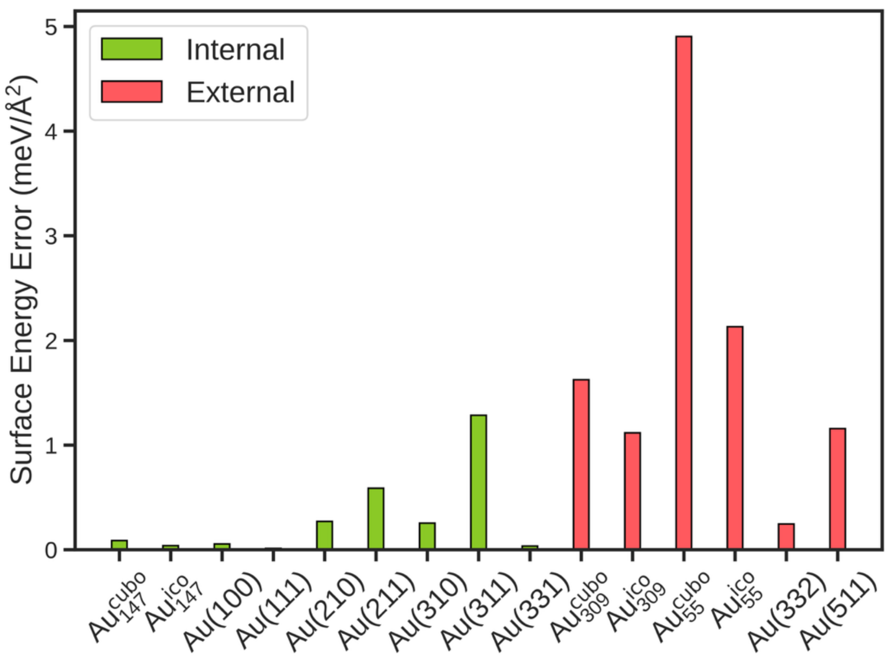

Figure S5. Errors in predicting the surface energies for the internal and external validation tests. Internal (external) validation tests refer to configurations collected by FLARE where their input structure is (not) used in constructing the training set.

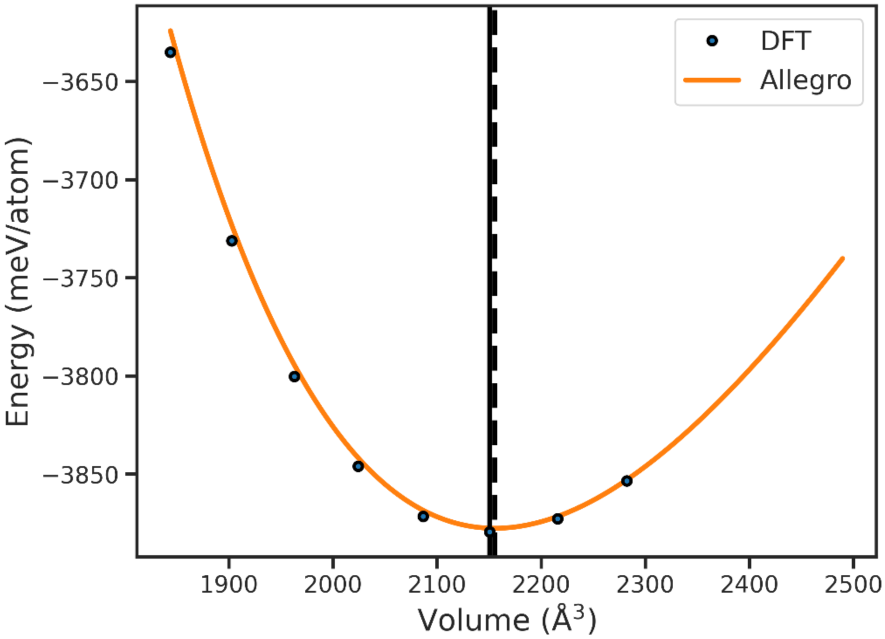

Figure S6. Total energy *versus* volume of a 5×5×5 unit cell of bulk Au at various strains. The solid (dashed) vertical line corresponds to the equilibrium volume from DFT (Allegro).

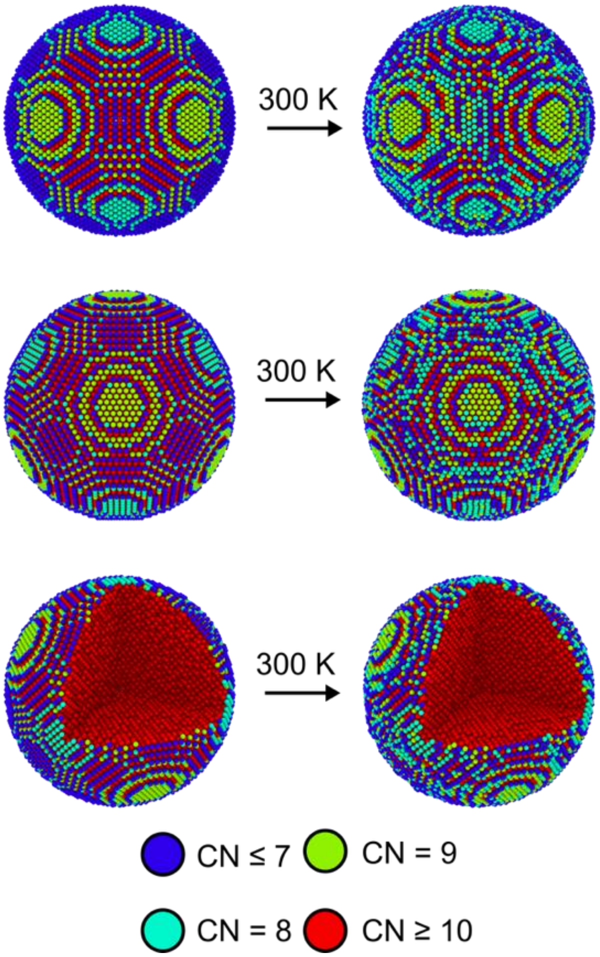

Figure S7. Additional views of the Au nanosphere in the ideal geometry (left column) and after thermal equilibration + 60 ps (right column)

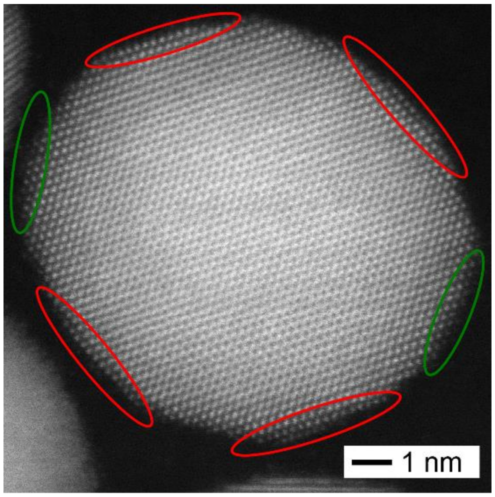

Figure S8. HAADF-STEM image of a 12-nm $Au@Pt_{1L}$ sphere. The circles indicate the regions where interatomic distances were measured to resolve the core-shell structure (red: [111] direction, green: [100] direction). The results are summarized in Table S5.

Table S5. Summary of the interatomic distances measured on 12-nm $Au@Pt_{1L}$ and Au spheres along [111] and [100] directions.

Interatomic distance across 5 atoms on the nth atomic layer along [111] direction		
	1st atomic layer	2nd atomic layer
$Au@Pt_{1L}$ nanospheres	1.18 $\pm$ 0.01 nm	1.24 $\pm$ 0.01 nm
Au nanospheres	1.23 $\pm$ 0.02 nm	1.23 $\pm$ 0.03 nm
Interatomic distance across 4 atoms on the nth atomic layer along [100] direction		
	1st atomic layer	2nd atomic layer
$Au@Pt_{1L}$ nanospheres	1.12 $\pm$ 0.01 nm	1.17 $\pm$ 0.01 nm
Au nanospheres	1.18 $\pm$ 0.01 nm	1.17 $\pm$ 0.01 nm
Measured lattice mismatch between Pt and Au	4.0 %	
Theoretical lattice mismatch between Pt and Au	4.2 %	

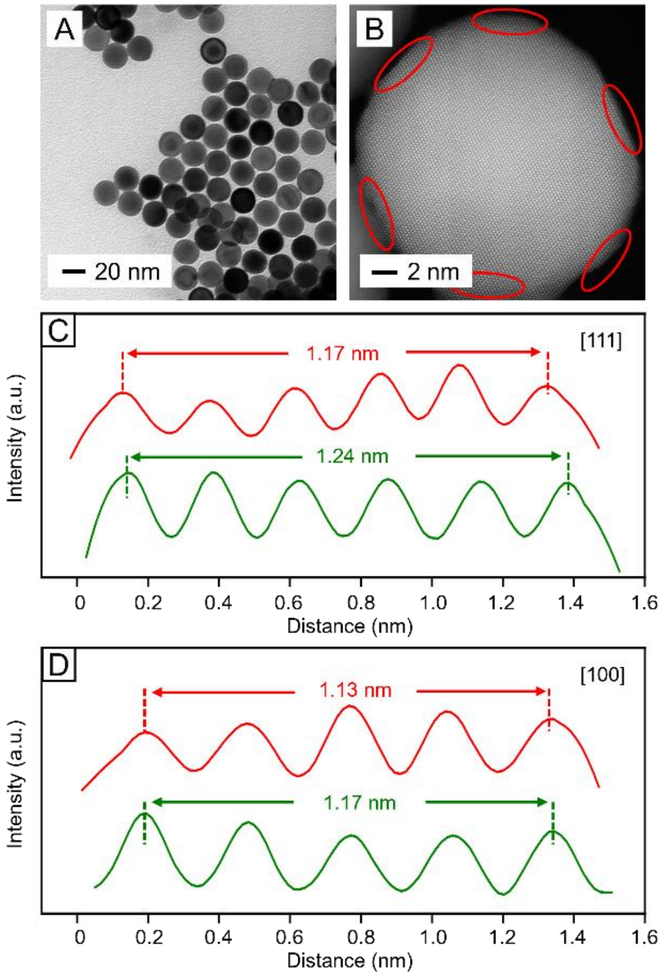

Figure S9. (A) TEM and (B) HAADF-STEM images of the 21-nm Au spheres after Pt deposition. The circles indicate the regions where interatomic distances were measured to resolve the core-shell structure. (C, D) Atomic intensity profiles measured along (C) [111] and (D) [100] directions, respectively, in the STEM images (red and green: the first and second layer of 21-nm $Au@Pt_{1L}$ nanosphere, respectively).

Table S6. Summary of the interatomic distances measured on 21-nm $Au@Pt_{1L}$ spheres along [111] and [100] directions.

Distance of 5 consecutive atoms on the nth atomic layer along [111] direction		
	1st atomic layer	2nd atomic layer
$Au@Pt_{1L}$ nanospheres	1.19 $\pm$ 0.02 nm	1.25 $\pm$ 0.01 nm
Distance of 4 consecutive atoms on the nth atomic layer along [100] direction		
	1st atomic layer	2nd atomic layer
$Au@Pt_{1L}$ nanospheres	1.13 $\pm$ 0.01 nm	1.18 $\pm$ 0.01 nm

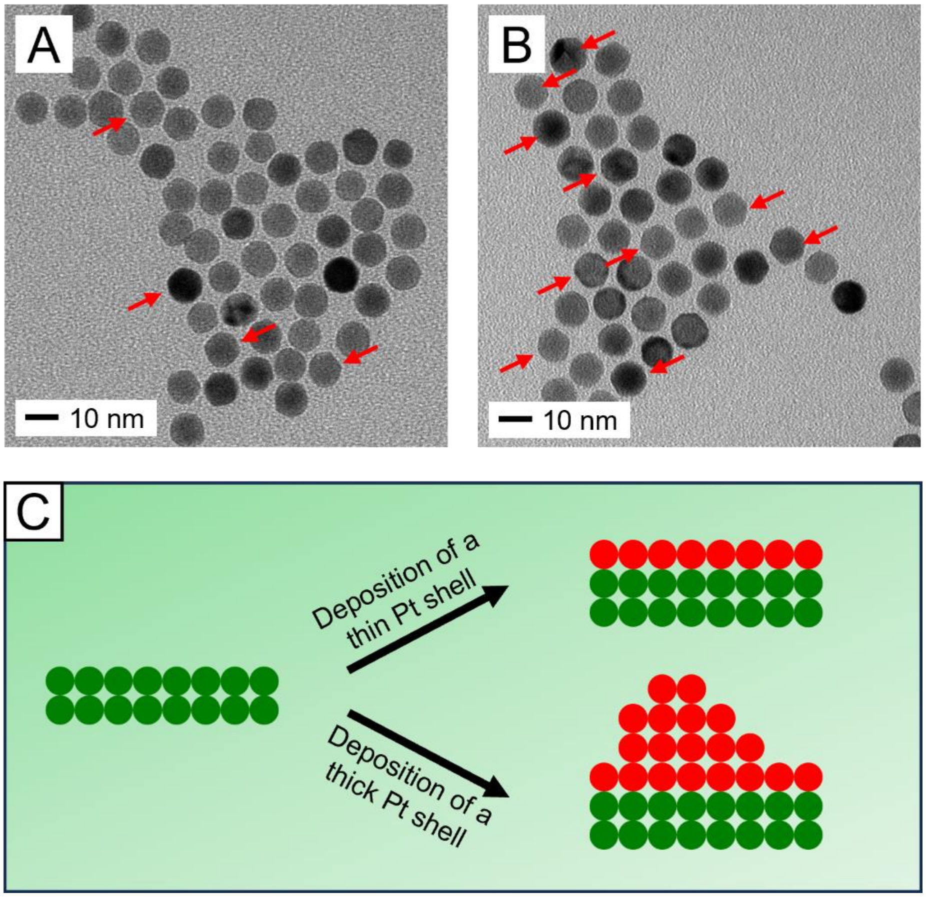

Figure S10. TEM images of the nanoparticles prepared by increasing the amount of Pt(II) precursor used for depositing Pt on the same batch of 12-nm Au spheres: (A) two- and (B) four-fold increase relative to the standard protocol. (C) Schematic of the differences between the final products obtained through layer-by-layer (upper route) and island (lower route) growth on the surface of Au nanospheres.

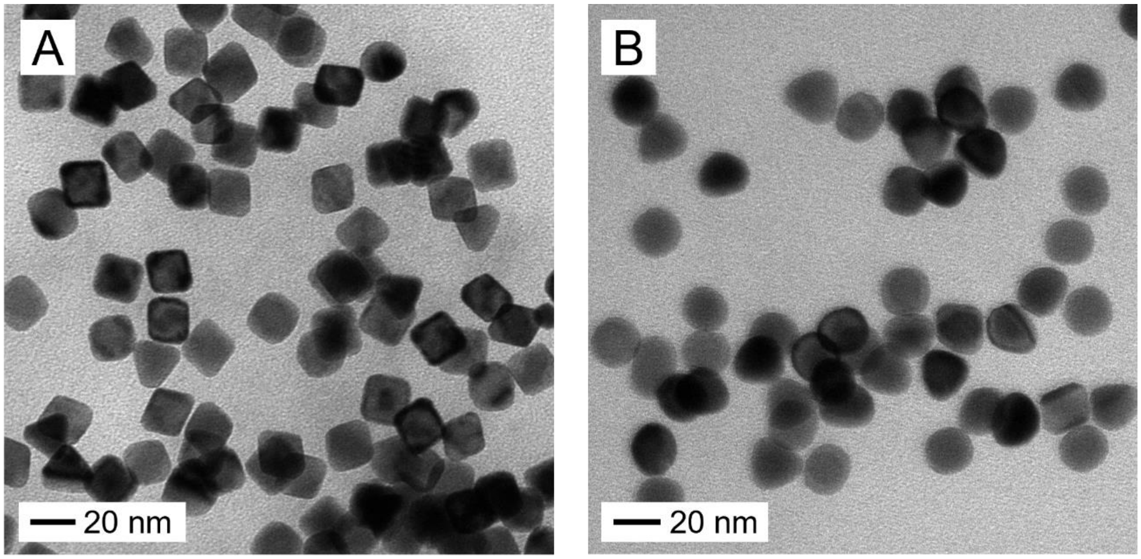

Figure S11. TEM images of (A) 20-nm Au@Pt octahedra and (B) 22-nm Au@Pt cuboctahedra.

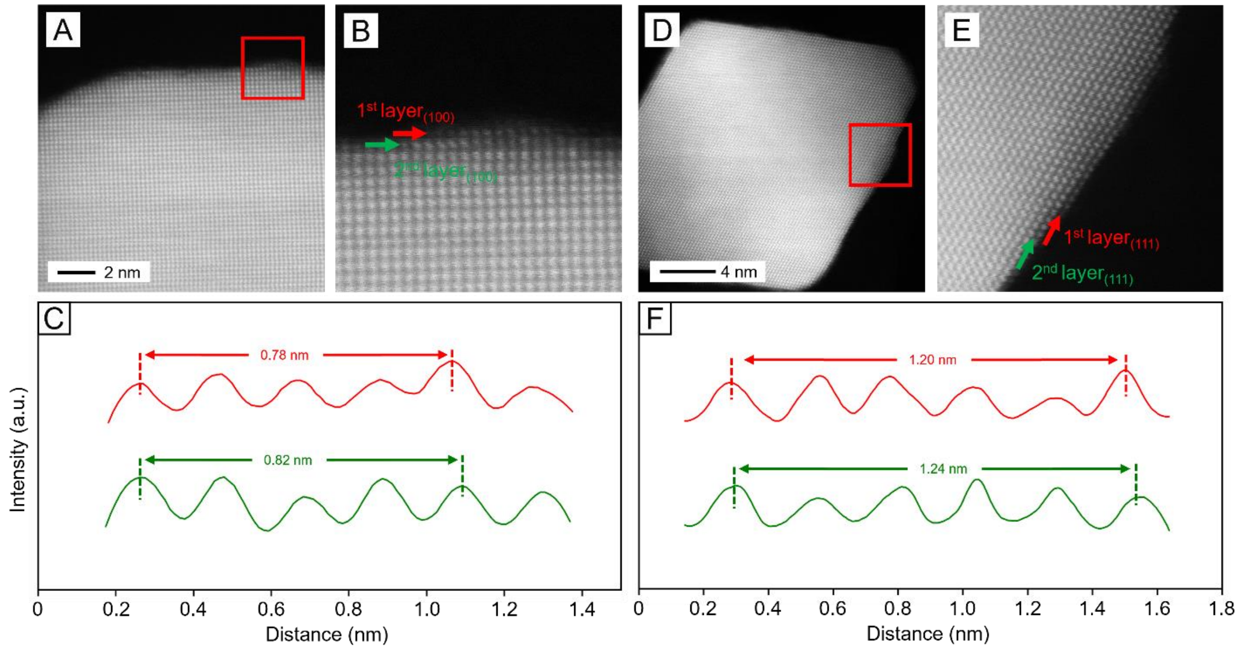

Figure S12. (A) HAADF-STEM image of a 22-nm Au@$Pt_{0.77L}$ cube. (B) A magnified region boxed in (A). (C) Atomic intensity profiles measured along different arrows in the STEM images (red and green: the first and second layers, respectively, of 22-nm Au@$Pt_{0.77L}$ cube along [100] direction). (D) HAADF-STEM image of a 20-nm Au@$Pt_{0.58L}$ octahedron. (E) A magnified region boxed in (D). (F) Atomic intensity profiles measured along different arrows in the STEM images (red and green: the first and second layers, respectively, of 20-nm Au@$Pt_{0.58L}$ octahedron along [111] direction).

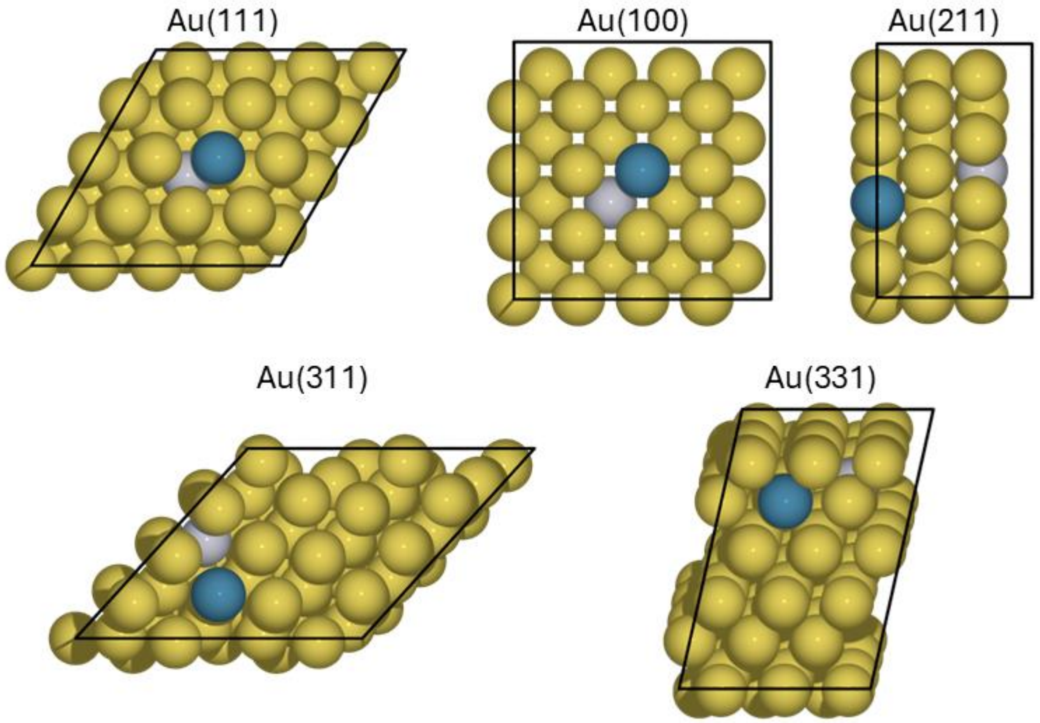

Figure S13. Illustrations depicting the top view of the slab models for Au(111), Au(100), Au(211), Au(311), and Au(331). Blue and grey spheres correspond to the preferred Au substitution site for Pt in the surface and subsurface, respectively. All spheres correspond to Au atoms.

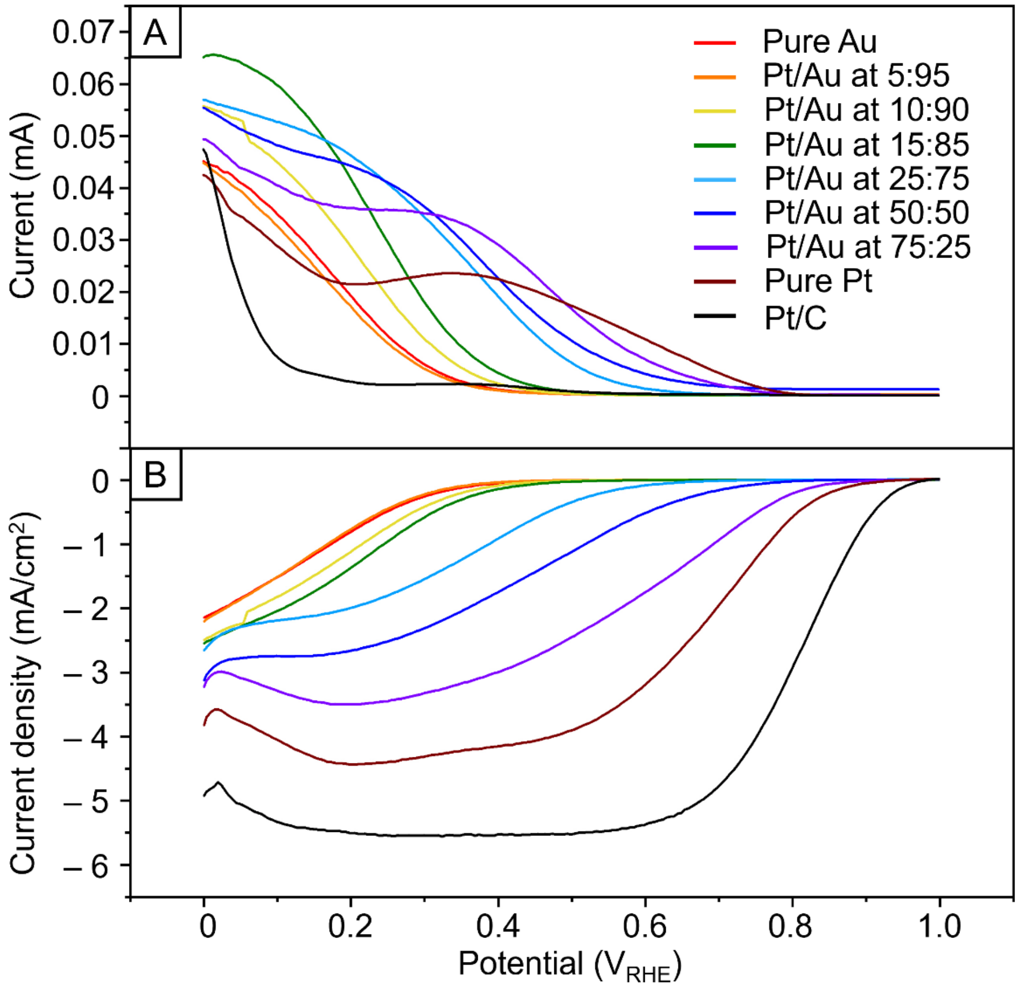

Figure S14. (A) Ring current *versus* disk potential, showing the activity toward H_2O_2 for the Au@Pt and Au@PtAu nanospheres synthesized with different feeding ratios of Pt(II) to Au(III) and (B) disk current *versus* disk potential, comparing the overall activity toward ORR.

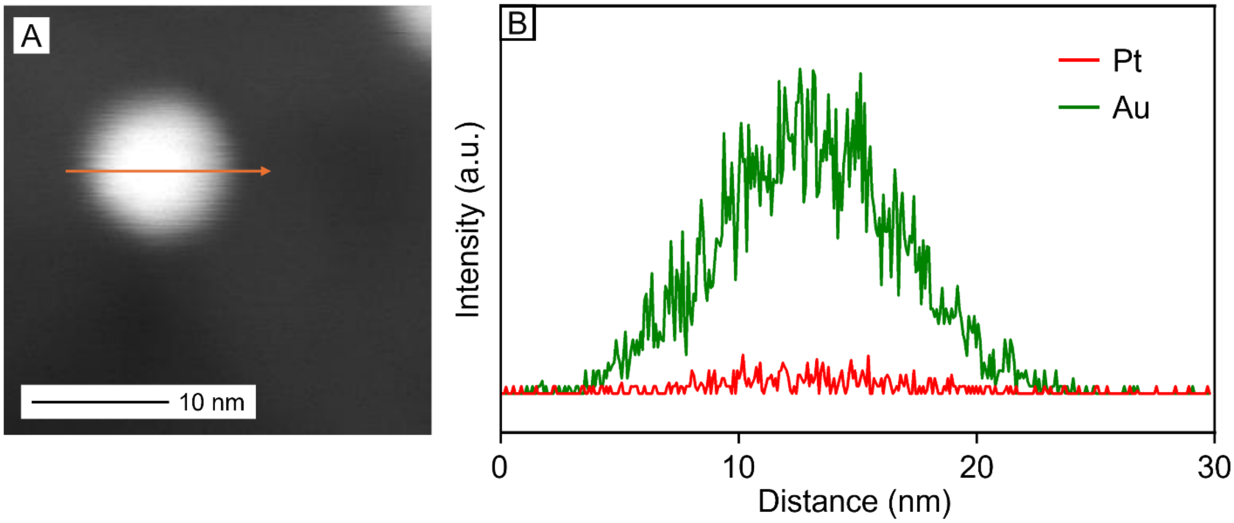

Figure S15. (A) HAADF-STEM image of a 12-nm Au@Pt_{1L} nanosphere after CV cycling for 20 rounds in O_2-saturated 0.1 M $HClO_4$. (B) EDX line scan of the particle. The Pt signal was concentrated in the core rather than uniformly distributed across the particle, indicating the alloying between the Pt atoms in the shell and the Au atoms in the core.

Table S7. Atomic ratio of Pt to Au in the final inks of Au@PtAu nanospheres synthesized with different feeding ratio of Pt(II) to Au(III).

Feeding ratio of Pt(II) to Au(III)	Atomic ratio of Pt to Au in the final ink
Pure Au	0
5:95	0.8:99.2
10:90	1.1:98.9
15:85	1.4:98.6
25:75	2.8:97.2
50:50	4.2:95.8
75:25	4.7:95.3
Pure Pt	6.2:93.8

REFENERCES

(1) Perdew, J. P.; Burke, K.; Ernzerhof, M. Generalized Gradient Approximation Made Simple. *Phys. Rev. Lett.* **1996**, *77*, 3865.

(2) Grimme, S.; Antony, J.; Ehrlich, S.; Krieg, H. A Consistent and Accurate *ab initio* Parametrization of Density Functional Dispersion Correction (DFT-D) for the 94 Elements H-Pu. *J. Chem. Phys.* **2010**, *132*, 154104.

(3) Kresse, G.; Furthmüller, Efficient Iterative Schemes for *ab initio* Total-Energy Calculations Using a Plane-Wave Basis Set. *J. Phys. Rev. B* **1996**, *54*, 11169.

(4) Kresse, G.; Joubert, D. From Ultrasoft Pseudopotentials to the Projector Augmented-Wave Method. *Phys. Rev. B* **1999**, *59*, 1758–1775.

(5) Kittel, C. *Introduction to Solid State Physics*, **2004**, *8th edition*, Wiley Sons, New York, NY.

(6) Haynes, W. M. (ed.), *CRC Handbook of Chemistry and Physics*, **2014**, *94th Edition*, CRC Press, Boca Raton, FL.

(7) Monkhorst, H. J.; Pack, J. D. Special Points for Brillouin-Zone Integrations. *Phys. Rev. B* **1976**, *13*, 5188.

(8) Larsen, A. H.; Mortensen, J. J.; Blomqvist, J.; Castelli, I. E.; Christensen, R.; Dułak, M.; Friis, J.; Groves, M. N.; Hammer, B.; Hargus, C. The Atomic Simulation Environment—A Python Library for Working with Atoms. *J. Phys. Condens. Matter.* **2017**, *29*, 273002.

(9) Bahn, S. R.; Jacobsen, K. W. An Object-Oriented Scripting Interface to a Legacy Electronic Structure Code. *CiSE* **2002**, *4*, 56–66.

(10) Tran, R.; Xu, Z.; Radhakrishnan, B.; Winston, D.; Sun, W.; Persson, K. A.; Ong, S. P. Surface Energies of Elemental Crystals. *Sci. Data* **2016**, *3*, 160080.

(11) Jain, A.; Ong, S. P.; Hautier, G.; Chen, W.; Richards, W. D.; Dacek, S.; Cholia, S.; Gunter, D.; Skinner, D.; Ceder, G.; Persson, K. A. Commentary: The Materials Project: A Materials Genome Approach to Accelerating Materials Innovation. *APL Mater.* **2013**, *1*, 011002.

(12) Vandermause, J.; Xie, Y.; Lim, J. S.; Owen, C. J.; Kozinsky, B. Active Learning of Reactive Bayesian Force Fields Applied to Heterogeneous Catalysis Dynamics of H/Pt. *Nat. Commun.* **2022**, *13*, 5183.

(13) Vandermause, J.; Torrisi, S. B.; Batzner, S.; Xie, Y.; Sun, L.; Kolpak, A. M.; Kozinsky, B. On-The-Fly Active Learning of Interpretable Bayesian Force Fields for Atomistic Rare Events. *Npj Comput. Mater.* **2020**, *6*, 20.

(14) Musaelian, A.; Batzner, S.; Johansson, A.; Sun, L.; Owen, C. J.; Kornbluth, M.;

Kozinsky, B. Learning Local Equivariant Representations for Large-Scale Atomistic Dynamics. *Nat. Commun.* **2023**, *14*, 579.

(15) Fiorentini, V.; Methfessel, M. Extracting Convergent Surface Energies from Slab Calculations. *J. Phys.: Condens. Matter.* **1996**, *8*, 6525.

(16) Stukowski, A. Computational Analysis Methods in Atomistic Modeling of Crystals. *JOM* **2014**, *66*, 399–407.

(17) Stukowski, A. Visualization and Analysis of Atomistic Simulation Data with OVITO–The Open Visualization Tool. *Modelling Simul. Mater. Sci. Eng.* **2009**, *18*, 015012.

(18) Thompson, A. P.; Aktulga, H. M.; Berger, R.; Bolintineanu, D. S.; Brown, W. M.; Crozier, P. S.; in 't Veld, P. J.; Kohlmeyer, A.; Moore, S. G.; Nguyen, T. D.; Shan, R.; Stevens, M. J.; Tranchida, J.; Trott, C.; Plimpton, S. J. LAMMPS - A Flexible Simulation Tool for Particle-Based Materials Modeling at the Atomic, Meso, and Continuum Scales. *Comput. Phys. Commun.* **2022**, *271*, 108171.

(19) Gissinger, J. R.; Nikiforov, I.; Afshar, Y.; Waters, B.; Choi, M.-K.; Karls, D. S.; Stukowski, A.; Im, W.; Heinz, H.; Kohlmeyer, A.; Tadmor, E. B. Type Label Framework for Bonded Force Fields in LAMMPS. *J. Phys. Chem. B* **2024**, *128*, 3282–3297

(20) Kozinsky, B.; Musaelian, A.; Johansson, A.; Batzner, S. Scaling the Leading Accuracy of Deep Equivariant Models to Biomolecular Simulations of Realistic Size. *SC '23: Proceedings of the International Conference for High Performance Computing, Networking, Storage and Analysis*, Denver CO USA; 979-8-4007-0109-2
